# Intelligent route to design efficient CO_2_ reduction electrocatalysts using ANFIS optimized by GA and PSO

**DOI:** 10.1038/s41598-022-25512-8

**Published:** 2022-12-02

**Authors:** Majedeh Gheytanzadeh, Alireza Baghban, Sajjad Habibzadeh, Karam Jabbour, Amin Esmaeili, Amin Hamed Mashhadzadeh, Ahmad Mohaddespour

**Affiliations:** 1 Surface Reaction and Advanced Energy Materials Laboratory, Chemical Engineering Department, Amirkabir University of Technology (Tehran Polytechnic), Tehran, Iran; 2 Chemical Engineering Department, Amirkabir University of Technology (Tehran Polytechnic), Mahshahr Campus, Mahshahr, Iran; 3 College of Engineering and Technology, American University of the Middle East, Kuwait City, Kuwait; 4 Department of Chemical Engineering, School of Engineering Technology and Industrial Trades, College of the North Atlantic - Qatar, Doha, Qatar; 5 Mechanical and Aerospace Engineering, School of Engineering and Digital Sciences, Nazarbayev University, 010000 Nur-Sultan, Kazakhstan

**Keywords:** Climate sciences, Environmental sciences, Chemistry, Engineering, Materials science, Nanoscience and technology

## Abstract

Recently, electrochemical reduction of CO_2_ into value-added fuels has been noticed as a promising process to decrease CO_2_ emissions. The development of such technology is strongly depended upon tuning the surface properties of the applied electrocatalysts. Considering the high cost and time-consuming experimental investigations, computational methods, particularly machine learning algorithms, can be the appropriate approach for efficiently screening the metal alloys as the electrocatalysts. In doing so, to represent the surface properties of the electrocatalysts numerically, *d*-band theory-based electronic features and intrinsic properties obtained from density functional theory (DFT) calculations were used as descriptors. Accordingly, a dataset containg 258 data points was extracted from the DFT method to use in machine learning method. The primary purpose of this study is to establish a new model through machine learning methods; namely, adaptive neuro-fuzzy inference system (ANFIS) combined with particle swarm optimization (PSO) and genetic algorithm (GA) for the prediction of *CO (the key intermediate) adsorption energy as the efficiency metric. The developed ANFIS–PSO and ANFIS–GA showed excellent performance with RMSE of 0.0411 and 0.0383, respectively, the minimum errors reported so far in this field. Additionally, the sensitivity analysis showed that the center and the filling of the *d*-band are the most determining parameters for the electrocatalyst surface reactivity. The present study conveniently indicates the potential and value of machine learning in directing the experimental efforts in alloy system electrocatalysts for CO_2_ reduction.

## Introduction

Transportation, power plants, and energy-intensive industries are some of the industrial activities that commonly emit greenhouse gases including CO_2_, CH_4_, and NO_x_. Around 75% of greenhouse gas emissions are carbon dioxide emissions^1^, which contribute to the 1.5 °C rise in global temperature that is considered to be a reasonably large amount. As a result, during the past 20 years, economic sustainability has gained international attention, drawing experts in the field of the environment, decision-makers, and international organizations from many nations. The United Nations Framework Convention on Climate Change was founded in 1992 as a result of this phenomena. The Kyoto Protocol and the 2015 Paris Agreement were later created in 1997 and 2015, respectively, to fight global warming by regulating greenhouse gas emissions^2^.

Nowadays, one of the significant challenges in the energy sector that should be effectively addressed is the development of sustainable energy resources. This is because the current energy conversion technologies cannot meet the energy requirement^3^. The electrochemical reduction of carbon dioxide is a necessary process that uses electricity from renewables (solar, wind, etc.) to produce fuels or value-added chemicals from water and CO_2_^4^. The *CO is the primary intermediate in the reduction of CO_2_. Indeed, as explained in Hori et al. study^5^, it is the only molecule having single carbon, which shows a product spectrum similar to CO_2_ on a copper electrode. In this process, the determining component in providing an efficient conversion relies on the electrocatalyst^6,7^. Several experiments and computations have been performed to find and prepare new materials as the electrocatalyst for the CO_2_ electroreduction^8–13^.

Therefore, understanding the surface chemical reactivity and the bond-breaking and -forming on the electrocatalyst’s surface is required to describe such a surface phenomenon. A significant volume of concepts has been established for the adsorption of the simple molecules onto the surface of transition-metal electrocatalysts^14–18^, among which the Sabatier principle is a main comprehensive concept^19^. It explains that the crucial reaction intermediate should have enough adsorption strength to increase the activity of the electrocatalyst; too strong or weak binding causes difficult product desorption and inadequate reactant activation, respectively. Thus, the reactivity as the function of binding energies can be described as a volcano-shaped plot^20^. Nørskov and co-workers, through a series of pioneering studies, claimed that the chemisorption of the adsorbate is severely dependent on the surface electronic structure (*d*-band theory^21^).

According to the *d*-band theory, the strength of the bond is given by the construction of the bonding and antibonding states, between the transition metal *d* states and the adsorbate valence states, and antibonding states energy comparative to the Fermi level (filling). The antibonding states are upper the *d* states, making the energy of the *d* states center a well initial representor of the bond strength. The greater *d*-band center in energy results in higher adsorbate–metal antibonding states, less occupation of antibonding states, and stronger adsorption bonds^22^. Although due to the recent improvements in electronic structure approaches (primarily density functional theory (DFT)), the *d*-band theory could provide understandings of the surface activity for the transition metals along with a minor group of alloys^3,23–29^, it could not explain the measured activity in some cases^30–32^. This could be probably since the spread in energy states was not considered. The definition of parameters such as the upper edge of the *d*-band (E_u_) and the *d*-band width (W_d_) by using Hilbert transform of the projected density of states (DOS) has improved the correlation between the activity and the *d*-band center^31^.

Inspired by the *d*-band chemisorption theory, the thought is to develop a computational relation between the electronic and intrinsic properties (descriptors) of clean surfaces and *CO adsorption energy. This helps to perform the material screening employing these descriptors, suppressing the costly and time-consuming experiments. While many linear relations between the *d*-band theory characteristics and the binding energy of the adsorbate have been published^31–33^, machine learning (ML) techniques were used in the present study in order to incorporate the possible nonlinear relation between the parameters, raise the accuracy of the prediction, and be more comprehensive.

ML techniques have been widely used to explore and investigate diverse phenomena in chemical engineering^34–41^. In the present study, Ma et al.^42^ predicted the *CO binding energy on various alloy systems using artificial neural networks (ANN) algorithm with root mean square error (RMSE) of 0.13 eV. In another study, Li et al.^43^ obtained almost the same accuracy (RMSE ∼ 0.12 eV) when using the same ML algorithm but geometric descriptors as inputs. Recently, Noh et al.^44^ proposed an ML model with RMSE of 0.05 eV. Unlike the other studies, they used two non-ab Initio input properties to forecast the binding energy of *CO in different systems. Since several aspects of an accurate model are still under question, it is necessary to establish a new model that enhances the prediction’s accuracy and decreases the model's uncertainty.

A fuzzy method is one of the soft computing methods which has a significant capacity in modeling complicated and nonlinear systems. Adaptive neuro-fuzzy inference system (ANFIS), a hybrid of ANN and the fuzzy inference system (FIS), can anticipate the nonlinear systems significantly^45–48^. The suggested model is based on the combination of genetic algorithm (GA) and particle swarm optimization (PSO) to ANFIS. To our knowledge, this investigation is the first attempt to apply ANFIS–GA and ANFIS–PSO in CO_2_ electroreduction process. In this research, the *CO adsorption energy in the CO_2_ electroreduction process was anticipated by the ANFIS–PSO and ANFIS–GA models. The PSO and GA were interconnected to ANFIS to balance the model’s complication and their generality capacity. Some intrinsic features and DFT-calculated electronic features of clean alloy surfaces, explicitly, parameters of the *d*-band distribution were used as inputs of the model.

## Methodology

### ANFIS background

**Figure 1:**
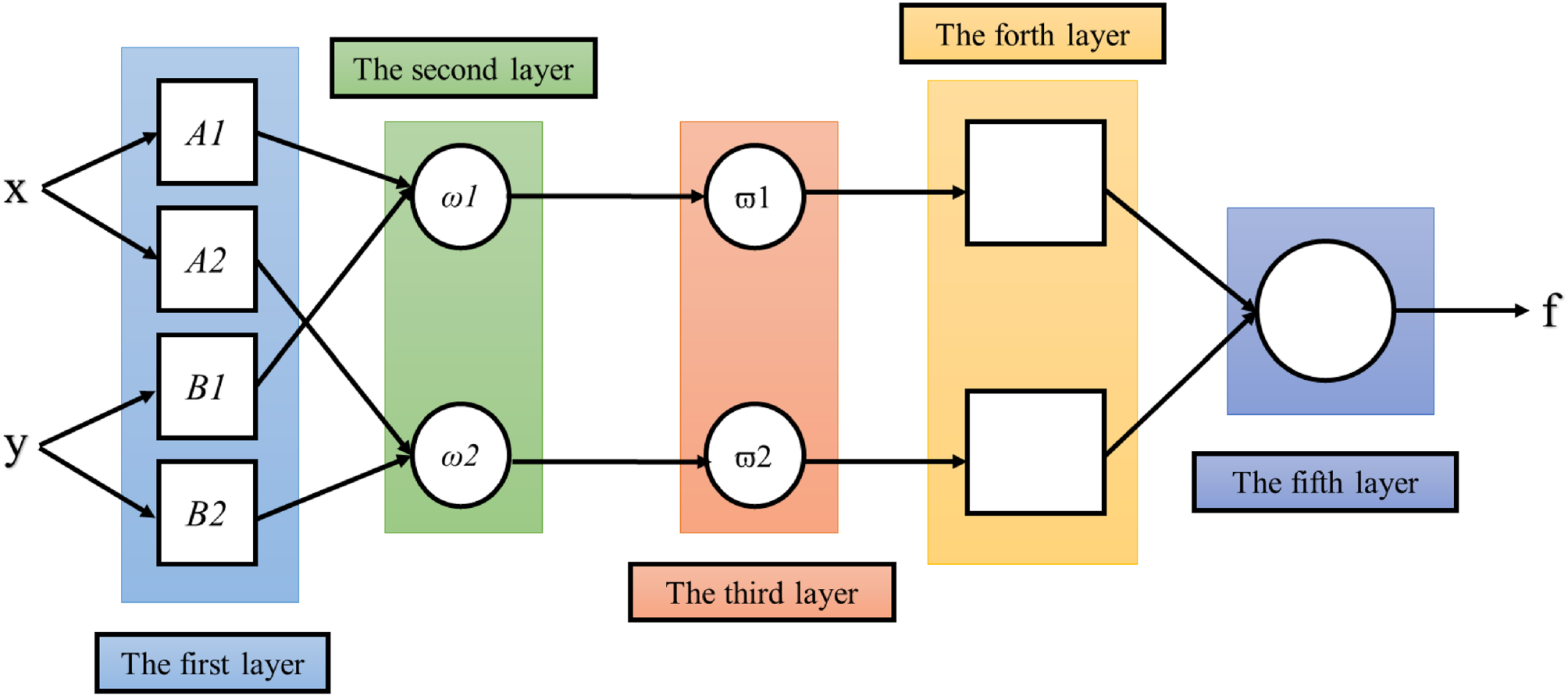
ANFIS structure.

The ANFIS structure used in this study includes five layers and thirteen inputs, where the Takagi–Sugeno system was employed as FIS. The fuzzy rules are presented as follows^46,49^:

$$ \begin{aligned} & {\text{Rule}}\,i: \\ & {\text{If}}\,x_{1} \,{\text{and}}\,x_{2} \,{\text{are}}\,A_{1} \,{\text{and}}\,B_{1} ,\,{\text{ respectively}},\,{\text{ then}}\,f_{i} = k_{1}^{i} x_{1} + k_{2}^{i} x_{2} + k_{0}^{i} \\ \end{aligned} $$

where *k*_*1*_, *k*_*2*_, and *k*_*0*_ represent the function parameters of output (*f*) and *A* and *B* indicate the membership functions for inputs (*x*_*1*_ and *x*_*2*_). The basic arrangement of ANFIS is a feedforward system that comprises five layers having several functions^50^ (Fig. 1). The learning structure of ANFIS is well discussed by several studies^51–53^.

The ANFIS with five layers is explained^54^:

In Layer 1, fuzzification, the whole of the nodes are supposed to be adaptive inputs.

1 $$ O_{i}^{1} = \mu A_{i} \left( {x_{1} } \right) \quad i = 1, 2, \ldots , n $$

2 $$ O_{i}^{1} = \mu B_{i - 2} \left( {x_{2} } \right) \quad i = 3, 4, \ldots , n $$

where *n* and both of $\mu {A}_{i}\left({x}_{1}\right)$ and $\mu {B}_{i-2}\left({x}_{2}\right)$ are the number of fuzzy sets per input variables and functions of Gaussian membership, respectively.

Layer 2, which is the product layer, the output of this layer is the product of the input signal based on:

3 $$ O_{i}^{2} = \omega_{i} = \mu A_{i} \left( {x_{1} } \right)\mu B_{i} \left( {x_{2} } \right)\quad \quad i = 1 \,or\, 2 $$

Layer 3, named the normalized layer, a circle node is used to represent each node. The normalization function is defined as:

4 $$ O_{i}^{3} = \overline{w}_{i} = \frac{{w_{i} }}{{w_{1} + w_{2} }}\quad \quad i = 1 \,or\, 2 $$

Layer 4, defuzzification, every node adapted with the following function:

5 $$ O_{i}^{4} = \overline{w}_{i} f_{i} = \overline{w}_{i} \left( {k_{1}^{i} x + k_{2}^{i} y + k_{0}^{i} } \right)\quad \quad i = 1 \,or\, 2 $$

where $({k}_{1}^{i}x+{k}_{2}^{i}y+{k}_{0}^{i})$ and ${\overline{w} }_{i}$ are the variable sets of ${\overline{w} }_{i}$’s node and the output of Layer 3, respectively.

In Layer 5, which is the output layer, summation of the arrival signals creates the output model:

6 $$ O_{i}^{5} = overall\, output = \sum\nolimits_{i} {\overline{w}_{i} f_{i} } = \frac{{\mathop \sum \nolimits_{i} w_{i} f_{i} }}{{\mathop \sum \nolimits_{i} w_{i} }}\quad \quad i = 1, 2 $$

Generally, an adaptive FIS comprises two diverse sections, the premise section and the consequent Section^47^, optimized with various methods. A hybrid learning optimization method is developed, including the least squares method and the gradient descent method. In the present study, the fuzzy c-means (FCM) process is interconnected to ANFIS algorithm to forecast *CO adsorption energy.

### FCM clustering

FCM is a method for data clustering where each point of data categorizes in clusters with different membership grades. FCM distributes a group of n vector ${x}_{i}$, $i=\mathrm{1,2},\dots ,n$ into fuzzy categories and find a center for the cluster in each category such that the cost function is minimized according to the dissimilarity value.

By membership matrix *U*, the cost function can be calculated as:

7 $$ J\left( {U,v_{1} , \ldots ,v_{K} } \right) = \sum\nolimits_{i = 1}^{n} {\sum\nolimits_{k = 1}^{k} {\left( {u_{ik} } \right)^{m} d^{2} \left( {x_{i} ,v_{K} } \right)} } $$

here ${v}_{K}$=${v}_{ka}$, k = 1, .., k, a = 1, …, p stands for the center of mass of kth cluster. In addition, m refers to the cluster' degree.

Algorithm should solve U and ${v}_{1},\dots ,{v}_{K}$ by minimizing Eqs. (8 and 9).

8 $$ u_{ik} = \frac{1}{{\mathop \sum \nolimits_{l = 1}^{K} \left( {\frac{{d \left( {x_{i} ,v_{k} } \right)}}{{d \left( {x_{i} ,W_{l} } \right)}}} \right)^{{\frac{2}{m} - 1}} }} $$

9 $$ v_{k} = \frac{{\mathop \sum \nolimits_{i = 1}^{n} \left( {U_{ik} } \right)^{m} x_{i} }}{{\mathop \sum \nolimits_{i = 1}^{n} \left( {U_{ik} } \right)^{m} }} $$

Minimizing task is carried out by the following cost function.

10 $$ J\left( U \right) = \sum\nolimits_{k = 1}^{K} {\left( {\frac{{\mathop \sum \nolimits_{i = 1}^{n} \mathop \sum \nolimits_{j = 1}^{n} \left( {\left( {u_{ik} } \right)^{m} \left( {u_{jk} } \right)^{m} d_{ij} } \right)}}{{2\mathop \sum \nolimits_{s = 1}^{n} \left( {u_{sk} } \right)^{m} }}} \right)} $$

Nikafshan Rad et al.^46^ presented the FCM algorithm in detail.

### PSO algorithm

**Figure 2:**
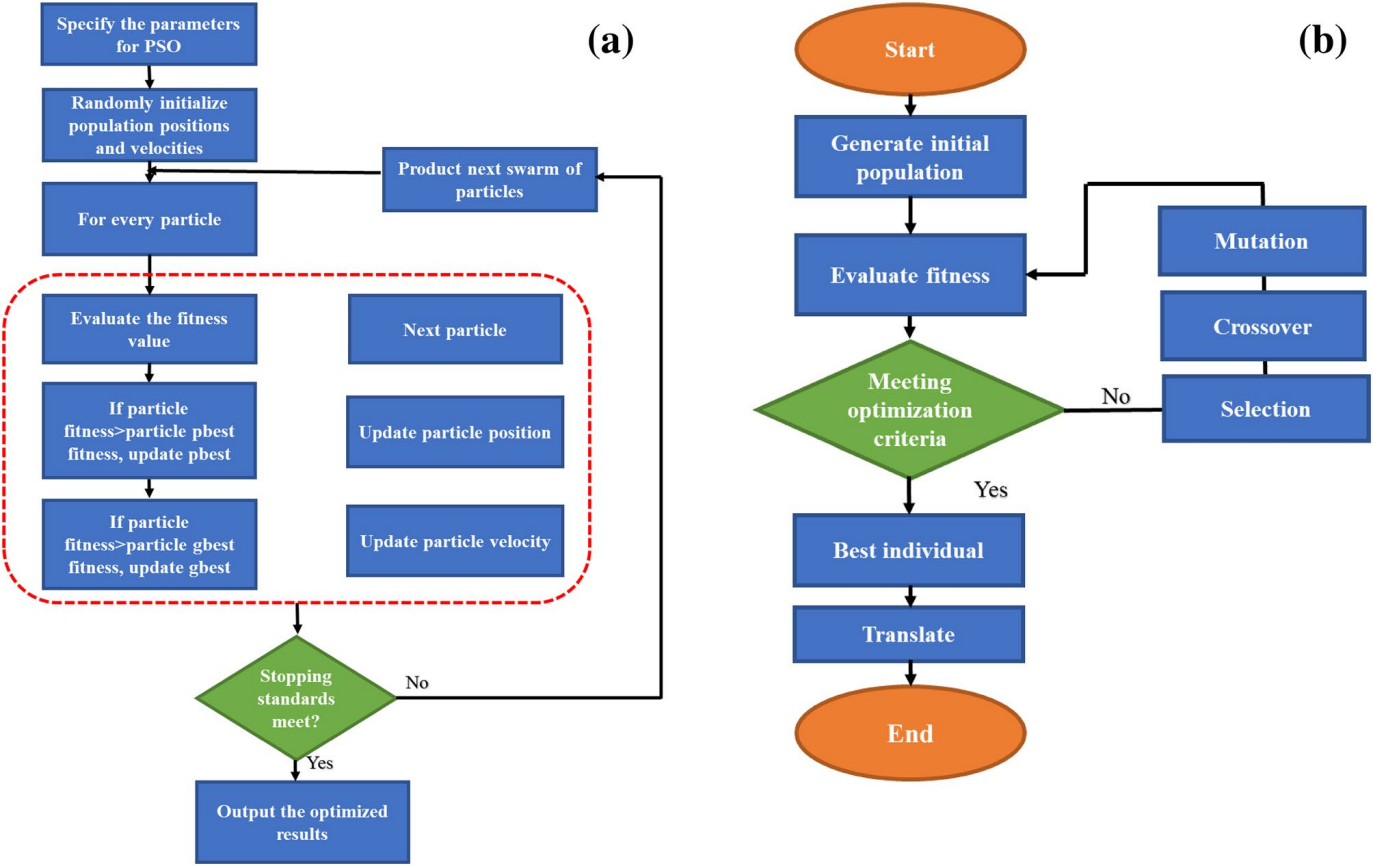
Scheme of the (**a**) PSO algorithm, (**b**) GA algorithm.

PSO is a population-based optimization approach that randomly selects a population of particles or solutions, then seeks optima through updating generation in each iteration^39^. Each particle is updated based on two particles: “pbest” and “gbest.” The “pbest” is the own best answer reached so far by particles, and the “gbest” is the total best solution obtained among the entire particles. Figure 2 shows the structure of PSO. In this scheme, random apportion velocities and positions are used at the first stage to start the initial population. The next step is to approximate each particle by regression analysis. When the stopping condition is satisfied by the best fitness rate of the particle, the process must be ended, and the factors should be reported. Suppose the fitness rate is not satisfactory for the ending criterion. In that case, the particles’ velocity and positions must be updated under two situations: In the first situation, if the particle fitness is larger than the gbest fitness, the related factors of gbest fitness should be updated. Second, if particle fitness is greater than the fitness of pbest, the pbest fitness factors should be updated. The other particles should be approximated through the second stage again.

### Genetic algorithm

GA is a general accidental exploration tool for optimization based on the ideas of natural selection and genetics. It uses probabilistic transition rules instead of definite ones, which results in capacity to investigate big solution spaces. GA includes three stages: (a) population initialization, (b) operators of GA (selection, crossover, and mutation), and (c) evaluation^49,55^.

1. *Population initialization:* In GA, each solution is named chromosome or string and is described via a series of different values. The strings (solutions) presenting each limitation or optimization issue necessities must be included in the initial population.
2. *Operators of GA:*
  1. *Selection:* It chooses solutions or individuals with high fitness values with a higher chance of surviving. A combination of Elitist and Tournament approaches is employed in this research through which the best solutions are chosen according to their fitness values and passed straight to the following generation.
  2. *Crossover:* In this one, two chosen individuals exchange part of their genes for making novel individuals for the following generation. Here, the scattered random method is used to create a new chromosome.
  3. *Mutation:* It makes a random change to the info within the strings or chromosomes. Occasionally, gene mutation occurs with a low possibility of transforming into new genes. The mutation operation increases the exploration ability of the search scheme to forbid to trap into local optima.
3. *Evaluation*: The function of fitness is applied to fit each individual solution.

The scheme of GA is illustrated in Fig. 2^49,56^.

## Modeling procedure

### Data collection

**Figure 3:**
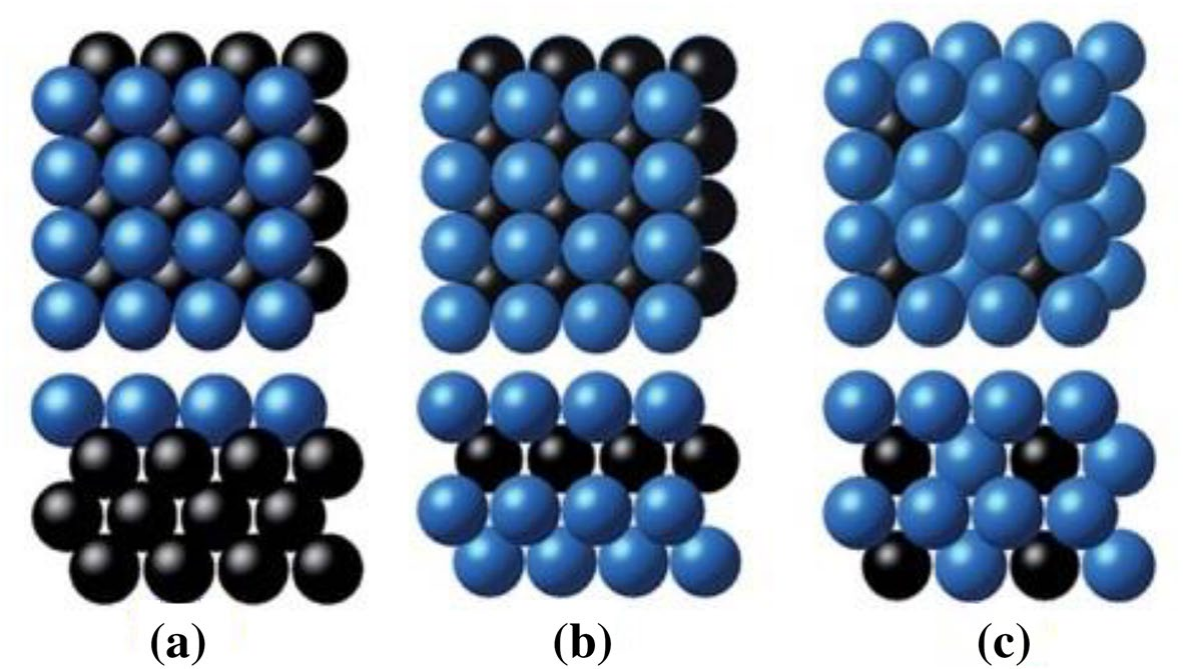
The {100}-terminated alloy surfaces in the form of (**a**) B@A, (**b**) AB@A, and (**c**) A3B@A. The first rows indicate the top view, and the second rows show the side view of the structures^42^.

In order to establish a relationship between the *CO binding energy and properties of electrocatalyst surfaces, a set of {100}-terminated bimetallic surfaces in the form of B@A, A-B@A, and A_3_B@A have been considered as illustrated in Fig. 3. In the B@A structure, group VIII and IB metals (Cu, Au, Ni, Ag, Pt, and Pd) were chosen for A and B, but in the other structures, B contains the post-transition and the d-block metals. The required properties as input parameters were obtained through geometry optimization in DFT calculations. The input parameters are classified into two groups: primary features, electronic properties of the d-states distribution, and secondary properties, which are physical constants of the host metal. The secondary properties were used for a well description of the chemical bonding on a sequences of metal surfaces. The width (square root of the 2nd central moment, *W*_d_), center (1st moment relative to the Fermi level, ε_d_), filling (zeroth moment up to the Fermi level, *f*), the local Pauling electronegativity (χ), skewness (3rd standardized moment, γ_1_), and kurtosis (4th standardized moment, γ_2_) are primary features while work function (*W*), ionization potential (IE), square of adsorbate–metal interatomic d coupling matrix element (V^2^_ad_), spatial extent of metal d-orbitals (*r*_*d*_), atomic radius (*r*_0_), Pauling electronegativity (χ_0_), and electron affinity (EA) are the secondary parameters. The 258 data points are taken from Ma et al. report^42^ and listed in Table S1. To develop the most precise model, randomly, 20% of the entire data was divided as the testing data to assay the model reliability and the remainder of them was used as the training data to study the *CO binding energy in CO_2_ electroreduction systems.

### Model evaluations

The statistical parameters (Eqs. 11–15) such as mean relative error (MRE), R^2^, mean-square error (MSE), the standard deviation (STD), root-mean-square error (RMSE) were used to assess the model accuracy.

11 $$ R^{2} = 1 - \frac{{\mathop \sum \nolimits_{i = 1}^{n} \left[ {x_{i}^{predicted} - x_{i}^{experimental} } \right]^{2} }}{{\mathop \sum \nolimits_{i = 1}^{n} \left[ {x_{i}^{predicted} - x_{m} } \right]^{2} }} $$

12 $$ STD = \sqrt {\mathop \sum \limits_{i = 1}^{n} \frac{{\left( {x_{i}^{predicted} - x_{m} } \right)^{2} }}{n}} $$

13 $$ MSE = \frac{1}{n}\mathop \sum \limits_{i = 1}^{n} \left( {x_{i}^{predicted} - x_{i}^{experimental} } \right)^{2} $$

14 $$ RMSE = \sqrt {\frac{{\mathop \sum \nolimits_{i = 1}^{n} \left( {x_{i}^{predicted} - x_{i}^{experimental} } \right)^{2} }}{n}} $$

15 $$ MRE = \frac{1}{n}\mathop \sum \limits_{i = 1}^{n} \frac{{\left| {x_{i}^{predicted} - x_{i}^{experimental} } \right|}}{{x_{i}^{experimental} }} $$

where n is the number of datapoints and m refers to mean value.

### Model development procedure

As mentioned above, there are 13 input variables to predict target parameter. In this study, 5 clusters with Gaussian membership functions were used for construction of ANFIS model. Gaussian function comprises of 2 paremeter that should be optimally determined during model development. Since there are 5 clusters and 13 inputs, there are 140 membership function parameters for optimization purpose. The optimum values of membership function parameters were determined by two evolutionary algorithms named PSO and GA. Setting of evolutionary algorithms was summarized in Table 1.

**Table 1:** Detail information of optimized ANFIS models.

ANFIS–PSO		ANFIS–GA	
No. of inputs	258	No. of inputs	258
No. of output	1	No. of output	1
Membership function	Gaussian	Membership function	Gaussian
No. of cluster	5	No. of cluster	5
No. of tuneing variables	140	No. of tuneing variables	140
Population size	45	Population size	45
Iteration	1500	Iteration	1500
C1	1		
C2	2		

### Accuracy of the collected data

**Figure 4:**
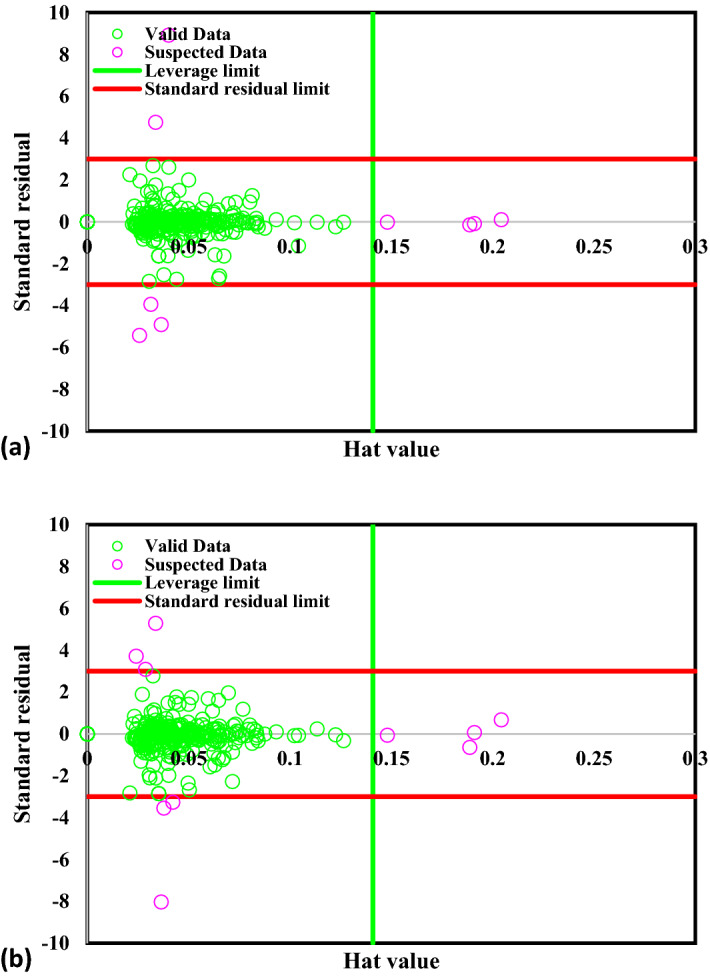
Detection of suspicious data for (**a**) PSO–ANFIS, (**b**) GA–ANFIS.

Some suspected data or outliers in the data bank show inconsistent behavior with the rest of the data. They are probably generated because of the degree of the accuracy of the calculation and assumptions (or instrumental and human errors in the case of experimental data banks). It is vital to distinguish them because they can cause the wrong prediction for the developed model^34,36^. To search the suspicious data and elevate the quality of data bank, the Leverage method is applied through which two parameters of critical leverage limit (H*) and Hat matrix (H) are calculated as follows:

16 $$ H = U\left( {U^{T} U} \right)^{ - 1} U^{T} $$

17 $$ H^{*} = \frac{3j}{{i + 1}} $$

where i, j, and U are the number of the model parameters, the number of training data, and a matrix dimensional of i * j, respectively. William’s plot, the standardized residuals versus Hat values, is depicted in Fig. 4. In this plot, the reliable zone is defined as the region bounded between the standardized residuals of − 3 to 3 and the critical leverage limit. According to Fig. 4, it can be clearly seen that most of the *CO binding energy values are placed in the valid area other than only 10 points among 258 data points, demonstrating the dataset is outstanding for training and testing models of ANFIS–PSO and ANFIS–GA.

## Results and discussion

### Analysis of sensitivity

**Figure 5:**
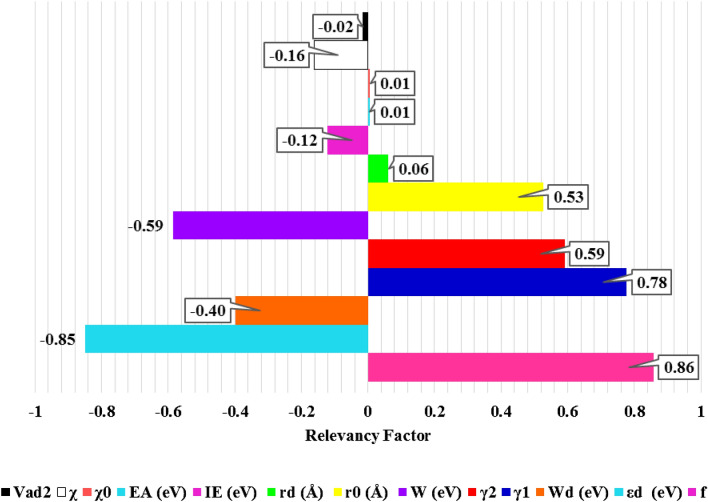
Analysis of sensitivity of the input parameters for *CO adsorption energy on the alloy surfaces.

Analysis of sensitivity is usually performed to study how the input parameters affect the output quantity^35^. In this analysis, relevancy factor (r) indicates the most influential input parameter on *CO binding energy, which is calculated as:

18 $$ r = \frac{{\mathop \sum \nolimits_{i = 1}^{n} \left( {X_{k.i} - \overline{X}_{k} } \right)\left( {Y_{i} - \overline{Y}} \right)}}{{\sqrt {\mathop \sum \nolimits_{i = 1}^{n} \left( {X_{k.i} - \overline{X}_{k} } \right)^{2} \mathop \sum \nolimits_{i = 1}^{n} \left( {Y_{i} - \overline{Y}} \right)^{2} } }} $$

where ${X}_{k.i}$, ${\overline{X} }_{k}$, ${Y}_{i}$, $\overline{Y }$, and n indicate the ‘k’ th input parameter, the average of the input parameters, ‘i’ th output, the outputs average, and the number of all data points, respectively. Generally, *r* value changes between − 1 to + 1. The more absolute value of r shows the greater effect of the corresponding input on the output for each input. The negative and positive values denote that the high input the less and more in the output, respectively ^57^. According to Fig. 5, the *CO binding energy (consequently the reactivity of the metal surface) has direct relationship with the filling of the *d*-band, skewness of the *d*-band, Kurtosis of a *d*-band, atomic radius, spatial extent of *d-*orbitals, electron affinity, and Pauling electronegativity and has an inverse relationship with the center of the *d*-band, width of a *d*-band, work function, ionization potential, square of adsorbate–metal interatomic *d* coupling matrix element, and local Pauling electronegativity.

As expected from the *d*-band theory, the sensitivity analysis declared the filling and the center of the *d*-band are the most effective parameters by 0.86 and − 0.85 relevancy factor, respectively. Also, the higher moments of the d-states distribution (such as skewness with r value of 0.78), which represent the *d*-band shape play a relatively important role in determining the reactivity of the metal surface. On the other hand, both of the electron affinity and the Pauling electronegativity have minimum effect on the *CO binding energy with r value of 0.01.

It is worth mention that parameters such as electron affinity and the Pauling electronegativity are intrinsic properties of active metal atoms which alter slightly across the periodic table but the most influencing parameters such as *d*-band center can be adjusted by ligand and strain engineering^42^.

### Modeling results

To evaluate how precisely the suggested ANFIS–GA and ANFIS–PSO models are, the statistical factors are applied to determine the accordance between the data points and the anticipated values of the *CO binding energy, reported in Table 2. The ANFIS–GA and ANFIS–PSO models predicted the train data significantly excellent with R^2^ of 0.994 and 0.995, respectively. The relative error quantities demonstrate the high precision of the proposed models in data training; in particular, the RMSE values of 0.0383 and 0.0411 for ANFIS–GA and ANFIS–PSO, respectively, are lower than the previous reports (0.13^42^, 0.12^43^, and 0.05^44^). This indicates the strength of the proposed models, which used the *d*-band theory electronic properties and the intrinsic features as inputs, the same data trained in^42^ with RMSE of 0.13. In addition to the forecast precision of the training data, the capability of the developed models to predict the unseen *CO binding energy data points is critically important. Therefore, both of the established models were investigated for the testing data. Again, both of the ANFIS–GA and ANFIS–PSO models show close and great accuracy for prediction of the unseen *CO binding energy data, where MRE, R^2^, MSE, STD, RMSE and are 3.521%/6.520%, 0.993/0.993, 0.00180/0.00177, 0.0322/0.0295, 0.0425/0.0421and for ANFIS–PSO and ANFIS–GA algorithms, respectively.

**Table 2:** The statistical parameters of proposed ANFIS-GA and ANFIS-PSO models.

Model	Set	R^2^	MRE (%)	MSE	RMSE	STD
ANFIS–PSO	Train	0.994	5.621	0.001688655	0.0411	0.0283
	Test	0.993	3.521	0.001807063	0.0425	0.0322
	Total	0.994	5.099	0.001718058	0.0425	0.0293
ANFIS–GA	Train	0.995	5.534	0.001467699	0.0383	0.0268
	Test	0.993	6.520	0.001770905	0.0421	0.0295
	Total	0.994	5.779	0.001542992	0.0421	0.0275

**Figure 6:**
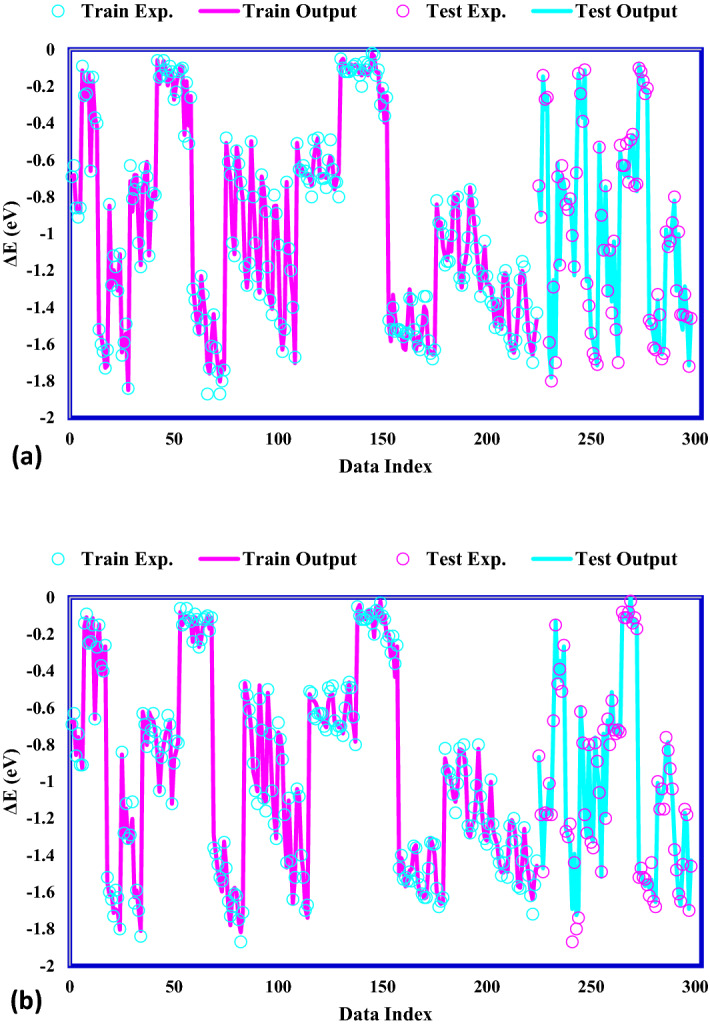
Comparison of actual and estimated values for (**a**) ANFIS–PSO, (**b**) ANFIS–GA.

For further confirmation the accuracy of the models, the data points and the anticipated values of the *CO binding energy are depicted by data indices in Fig. 6. As can be observed, the excellent agreement between the actual and estimated *CO binding energy amounts proves the excellent function of the established models. For both the ANFIS–GA and ANFIS–PSO models, the predicted values lines follow the actual data points precisely, which means these models have incredible capability to assess the reactivity of the metal alloy surfaces in terms of *CO adsorption energy.

**Figure 7:**
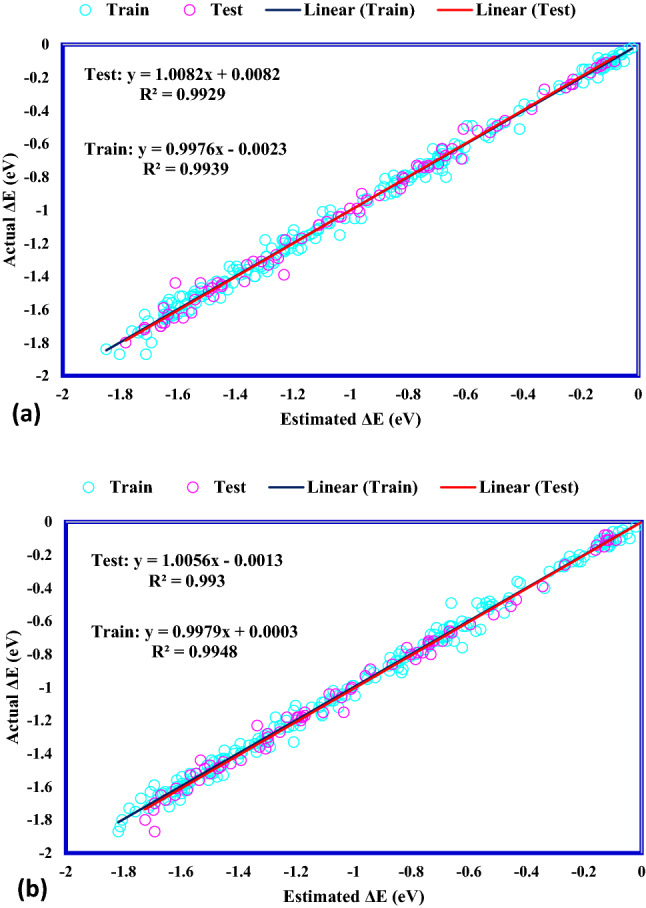
Cross plots for (**a**) ANFIS–PSO, (**b**) ANFIS–GA.

The forecasted *CO binding energy values versus actual data values are depicted in Fig. 7. For both of the proposed ANFIS–GA and ANFIS–PSO models, the predicted data are precisely placed on their actual values so that their linear fitting shows correlation coefficients of more than 0.99. The 45° line is a criterion for the accuracy of the suggested models, where as shown in Fig. 7, the drawn fitting lines cross it precisely.

**Figure 8:**
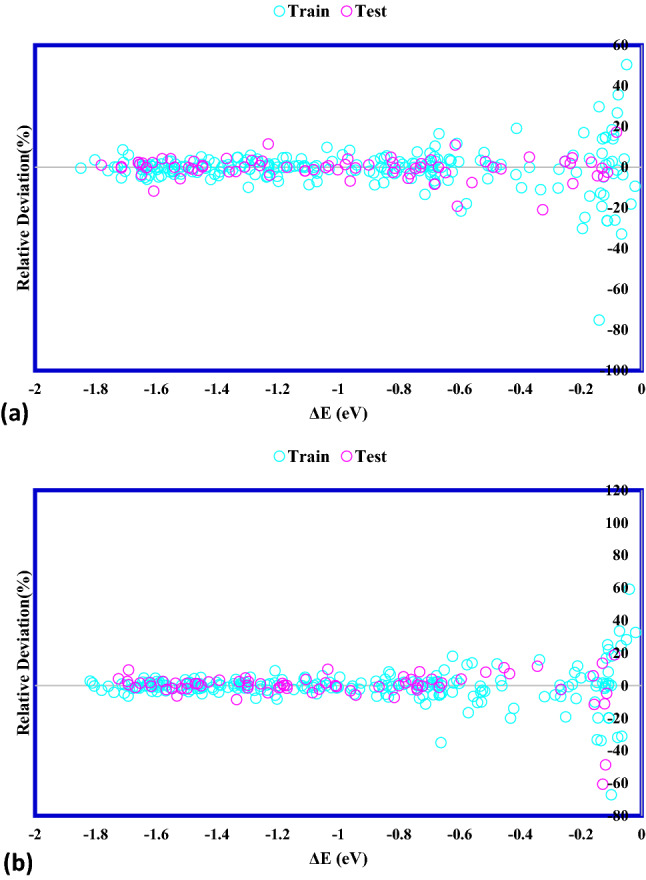
Comparison of experimental values and model outputs for (**a**) ANFIS–PSO, (**b**) ANFIS–GA.

Moreover, the percentage of relative deviations between actual and estimated *CO binding energy data is shown in Fig. 8 for testing and training data sets of the developed ANFIS–GA and ANFIS–PSO models. The percentage mean relative deviations of the training and testing data obtained by the PSO–ANFIS model are 5.621% and 3.521%, respectively. Also, these values were 5.534% and 6.520% for training and testing datasets of ANFIS–GA model, respectively.

## Conclusions

The present study conveniently indicates the potential and value of machine learning in directing the experimental efforts in alloy system electrocatalysts for CO2 reduction. Accordingly, two machine learning models of ANFIS–PSO and ANFIS–GA have been presented to anticipate the *CO binding energy on alloys electrocatalysts using electronic and intrinsic features. The required properties as input parameters were obtained through geometry optimization in DFT calculations.

The ANFIS–PSO and ANFIS–GA presented an excellent match between the *CO binding data and the predicted values with RMSE of 0.0411 and 0.0383, respectively, which is more precise than the other reported studies. In addition, it was found that the relative deviation of ANFIS-PSO model was 3.521%, while this value was 6.520% for the ANFIS–GA model, which indicates better ability of PSO to optimize ANFIS model. The percentage mean relative deviations of the training and testing data obtained by the PSO–ANFIS model are 5.621% and 3.521%, respectively. Also, these values were 5.534% and 6.520% for training and testing datasets of ANFIS–GA model, respectively. Moreover, the outlier detection technique was used to find outliers and it was shown by William's diagram. The sensitivity analysis confirmed the filling and the center of the *d*-band as the most influencing parameters for the electrocatalyst surface reactivity by 0.86 and − 0.85 relevancy factor, respectively. This study’s results and discussions can make it easier for scientists and chemical engineers to explore and examine new materials as electrocatalysts for the CO_2_ electroreduction process.

## Supplementary Information

Supplementary Information.

## Data Availability

All data generated or analysed during this study are included in this published article [and its supplementary information files].
