# Supplementary material for: Intelligent route to design efficient CO2 reduction electrocatalysts using ANFIS optimized by GA and PSO
Source: Sci Rep. 2022 Dec 2;12:20859. doi: 10.1038/s41598-022-25512-8 (PMC9718738; doi:10.1038/s41598-022-25512-8)
Supplement: Supplementary file 1 — Supplementary Information. [file 41598_2022_25512_MOESM1_ESM.docx]

**Intelligent Route to Design Efficient CO_2_ Reduction Electrocatalysts using ANFIS Optimized by GA and PSO**

Majedeh Gheytanzadeh^1^, Alireza Baghban^2,*^, Sajjad Habibzadeh^1,*^, Karam Jabbour^3^, Amin Esmaeili^4^, Amin Hamed Mashhadzadeh^5^ and Ahmad Mohaddespour^3^

*^1^ Surface reaction and advanced energy materials laboratory, Chemical Engineering Department, Amirkabir University of Technology (Tehran Polytechnic), Tehran, Iran*

*^2^ Chemical Engineering Department, Amirkabir University of Technology (Tehran Polytechnic), Mahshahr Campus, Mahshahr, Iran*

*^3^College of Engineering and Technology, American University of the Middle East, Kuwait*

*^4^Department of Chemical Engineering, School of Engineering Technology and Industrial Trades, College of the North Atlantic - Qatar, Doha, Qatar*

*^5^Mechanical and Aerospace Engineering, School of Engineering and Digital Sciences, Nazarbayev University, Nur-Sultan 010000, Kazakhstan*

**Corresponding authors: Alireza_baghban@alumni.ut.ac.ir (A. Baghban); sajjad.habibzadeh@mail.mcgill.ca (S.Habibzadeh)*

**Table S1:** Intrinsic features and DFT-calculated electronic properties as input

| f | εd (eV) | Wd (eV) | γ1 | γ2 | W (eV) | r0 (Å) | rd (Å) | IE (eV) | EA (eV) | χ0 | χ | Vad^2^ | ΔE (eV) |
| --- | --- | --- | --- | --- | --- | --- | --- | --- | --- | --- | --- | --- | --- |
| 0.97 | -2.21 | 1.11 | 1.08 | 8.87 | 5.3 | 1.41 | 0.67 | 7.73 | 1.24 | 1.9 | 1.9 | 1 | -0.69 |
| 0.97 | -2.25 | 1.05 | 1.13 | 10.94 | 5.3 | 1.41 | 0.67 | 7.73 | 1.24 | 1.9 | 1.9 | 1 | -0.63 |
| 0.98 | -1.66 | 0.97 | 0.47 | 8.6 | 5.3 | 1.41 | 0.67 | 7.73 | 1.24 | 1.9 | 2.03 | 1 | -0.86 |
| 0.98 | -1.9 | 0.88 | 0.06 | 15.15 | 5.3 | 1.41 | 0.67 | 7.73 | 1.24 | 1.9 | 1.91 | 1 | -0.74 |
| 0.97 | -2.1 | 1.13 | 0.26 | 8.42 | 5.3 | 1.41 | 0.67 | 7.73 | 1.24 | 1.9 | 2.03 | 1 | -0.91 |
| 0.97 | -1.85 | 1.09 | 0.03 | 8.18 | 5.3 | 1.41 | 0.67 | 7.73 | 1.24 | 1.9 | 2.06 | 1 | -0.91 |
| 0.98 | -1.98 | 1.02 | -0.42 | 10.94 | 5.3 | 1.41 | 0.67 | 7.73 | 1.24 | 1.9 | 2.16 | 1 | -0.86 |
| 0.98 | -3.64 | 1.22 | 1.97 | 11.27 | 5.01 | 1.59 | 0.89 | 7.58 | 1.3 | 1.93 | 2.05 | 2.26 | -0.14 |
| 0.98 | -3.92 | 1.1 | 2.38 | 16.87 | 5.01 | 1.59 | 0.89 | 7.58 | 1.3 | 1.93 | 1.93 | 2.26 | -0.09 |
| 0.98 | -3.81 | 1.36 | 1.65 | 9.62 | 5.01 | 1.59 | 0.89 | 7.58 | 1.3 | 1.93 | 2.05 | 2.26 | -0.25 |
| 0.98 | -3.69 | 1.28 | 1.75 | 10.39 | 5.01 | 1.59 | 0.89 | 7.58 | 1.3 | 1.93 | 2.08 | 2.26 | -0.24 |
| 0.98 | -3.87 | 1.16 | 1.93 | 13.38 | 5.01 | 1.59 | 0.89 | 7.58 | 1.3 | 1.93 | 2.18 | 2.26 | -0.15 |
| 0.96 | -3.88 | 1.9 | 0.85 | 5.14 | 6.01 | 1.59 | 1.01 | 9.23 | 2.31 | 2.54 | 2.23 | 3.35 | -0.66 |
| 0.97 | -3.07 | 1.52 | 1.02 | 5.86 | 6.01 | 1.59 | 1.01 | 9.23 | 2.31 | 2.54 | 2.38 | 3.35 | -0.27 |
| 0.97 | -3.21 | 1.34 | 1.22 | 8.47 | 6.01 | 1.59 | 1.01 | 9.23 | 2.31 | 2.54 | 2.25 | 3.35 | -0.15 |
| 0.96 | -3.41 | 1.72 | 0.89 | 5.32 | 6.01 | 1.59 | 1.01 | 9.23 | 2.31 | 2.54 | 2.38 | 3.35 | -0.37 |
| 0.97 | -3.17 | 1.59 | 0.91 | 5.62 | 6.01 | 1.59 | 1.01 | 9.23 | 2.31 | 2.54 | 2.42 | 3.35 | -0.4 |
| 0.97 | -3.18 | 1.41 | 0.92 | 7.01 | 6.01 | 1.59 | 1.01 | 9.23 | 2.31 | 2.54 | 2.54 | 3.35 | -0.26 |
| 0.87 | -1.32 | 1.33 | 0.36 | 5.23 | 5.77 | 1.38 | 0.71 | 7.64 | 1.16 | 1.91 | 1.91 | 1.16 | -1.52 |
| 0.88 | -1.01 | 1.22 | 0.39 | 7.1 | 5.77 | 1.38 | 0.71 | 7.64 | 1.16 | 1.91 | 1.91 | 1.16 | -1.6 |
| 0.87 | -1.13 | 1.22 | 0.06 | 5.79 | 5.77 | 1.38 | 0.71 | 7.64 | 1.16 | 1.91 | 2.03 | 1.16 | -1.64 |
| 0.88 | -0.77 | 1.05 | -0.37 | 11.06 | 5.77 | 1.38 | 0.71 | 7.64 | 1.16 | 1.91 | 1.92 | 1.16 | -1.73 |
| 0.87 | -1.21 | 1.36 | -0.31 | 6.48 | 5.77 | 1.38 | 0.71 | 7.64 | 1.16 | 1.91 | 2.03 | 1.16 | -1.59 |
| 0.88 | -1.24 | 1.34 | -0.28 | 6.05 | 5.77 | 1.38 | 0.71 | 7.64 | 1.16 | 1.91 | 2.07 | 1.16 | -1.63 |
| 0.88 | -0.87 | 1.19 | -0.8 | 9.02 | 5.77 | 1.38 | 0.71 | 7.64 | 1.16 | 1.91 | 2.17 | 1.16 | -1.8 |
| 0.93 | -2.05 | 1.62 | 0.25 | 3.27 | 5.9 | 1.52 | 0.94 | 8.34 | 0.56 | 2.2 | 2.06 | 2.78 | -0.84 |
| 0.92 | -1.67 | 1.36 | 0.23 | 3.93 | 5.9 | 1.52 | 0.94 | 8.34 | 0.56 | 2.2 | 2.2 | 2.78 | -1.28 |
| 0.93 | -1.31 | 1.23 | 0.02 | 6.87 | 5.9 | 1.52 | 0.94 | 8.34 | 0.56 | 2.2 | 2.08 | 2.78 | -1.12 |
| 0.91 | -2.15 | 1.6 | 0.14 | 3.98 | 5.9 | 1.52 | 0.94 | 8.34 | 0.56 | 2.2 | 2.2 | 2.78 | -1.18 |
| 0.92 | -1.82 | 1.48 | 0.05 | 4.32 | 5.9 | 1.52 | 0.94 | 8.34 | 0.56 | 2.2 | 2.24 | 2.78 | -1.31 |
| 0.93 | -1.35 | 1.35 | -0.21 | 5.94 | 5.9 | 1.52 | 0.94 | 8.34 | 0.56 | 2.2 | 2.35 | 2.78 | -1.29 |
| 0.89 | -2.33 | 2.05 | 0.14 | 2.75 | 6.74 | 1.53 | 1.04 | 9 | 2.13 | 2.28 | 2.1 | 3.9 | -1.11 |
| 0.88 | -1.9 | 1.74 | 0.27 | 3.46 | 6.74 | 1.53 | 1.04 | 9 | 2.13 | 2.28 | 2.24 | 3.9 | -1.66 |
| 0.9 | -1.38 | 1.52 | 0.14 | 5.03 | 6.74 | 1.53 | 1.04 | 9 | 2.13 | 2.28 | 2.12 | 3.9 | -1.59 |
| 0.88 | -2.37 | 2.04 | 0.21 | 3.35 | 6.74 | 1.53 | 1.04 | 9 | 2.13 | 2.28 | 2.24 | 3.9 | -1.49 |
| 0.88 | -2.03 | 1.87 | 0.17 | 3.65 | 6.74 | 1.53 | 1.04 | 9 | 2.13 | 2.28 | 2.28 | 3.9 | -1.7 |
| 0.9 | -1.46 | 1.64 | -0.06 | 4.52 | 6.74 | 1.53 | 1.04 | 9 | 2.13 | 2.28 | 2.39 | 3.9 | -1.84 |
| 0.97 | -2.72 | 0.99 | 2.79 | 16.86 | 5.3 | 1.41 | 0.67 | 7.73 | 1.24 | 1.9 | 1.64 | 1 | -0.63 |
| 0.97 | -2.83 | 1.02 | 2.48 | 14.5 | 5.3 | 1.41 | 0.67 | 7.73 | 1.24 | 1.9 | 1.73 | 1 | -0.69 |
| 0.97 | -2.74 | 1.07 | 2.12 | 11.81 | 5.3 | 1.41 | 0.67 | 7.73 | 1.24 | 1.9 | 1.77 | 1 | -0.81 |
| 0.97 | -2.59 | 1.1 | 1.71 | 9.68 | 5.3 | 1.41 | 0.67 | 7.73 | 1.24 | 1.9 | 1.79 | 1 | -1.17 |
| 0.97 | -2.34 | 1.08 | 1.28 | 9.6 | 5.3 | 1.41 | 0.67 | 7.73 | 1.24 | 1.9 | 1.89 | 1 | -0.76 |
| 0.97 | -2.14 | 1.07 | 1.25 | 9.36 | 5.3 | 1.41 | 0.67 | 7.73 | 1.24 | 1.9 | 1.9 | 1 | -0.72 |
| 0.97 | -2.26 | 1.06 | 1.11 | 10.84 | 5.3 | 1.41 | 0.67 | 7.73 | 1.24 | 1.9 | 1.9 | 1 | -0.63 |
| 0.97 | -2.56 | 0.96 | 2.76 | 17.57 | 5.3 | 1.41 | 0.67 | 7.73 | 1.24 | 1.9 | 1.56 | 1 | -0.73 |
| 0.97 | -2.74 | 1.02 | 2.58 | 15.81 | 5.3 | 1.41 | 0.67 | 7.73 | 1.24 | 1.9 | 1.62 | 1 | -0.84 |
| 0.97 | -2.71 | 1.08 | 2.14 | 12.69 | 5.3 | 1.41 | 0.67 | 7.73 | 1.24 | 1.9 | 1.76 | 1 | -1.05 |
| 0.97 | -2.52 | 1.11 | 1.58 | 9.93 | 5.3 | 1.41 | 0.67 | 7.73 | 1.24 | 1.9 | 2.01 | 1 | -1.18 |
| 0.97 | -2.31 | 1.08 | 0.94 | 8.48 | 5.3 | 1.41 | 0.67 | 7.73 | 1.24 | 1.9 | 2.03 | 1 | -0.87 |
| 0.97 | -2.14 | 1.07 | 0.85 | 8.58 | 5.3 | 1.41 | 0.67 | 7.73 | 1.24 | 1.9 | 2.06 | 1 | -0.81 |
| 0.97 | -1.99 | 1.03 | 0.71 | 9.38 | 5.3 | 1.41 | 0.67 | 7.73 | 1.24 | 1.9 | 2.03 | 1 | -0.75 |
| 0.98 | -2.13 | 0.98 | 0.68 | 14.09 | 5.3 | 1.41 | 0.67 | 7.73 | 1.24 | 1.9 | 1.91 | 1 | -0.64 |
| 0.97 | -2.41 | 0.93 | 2.54 | 19.64 | 5.3 | 1.41 | 0.67 | 7.73 | 1.24 | 1.9 | 1.49 | 1 | -0.69 |
| 0.97 | -2.83 | 1.14 | 2.06 | 12.45 | 5.3 | 1.41 | 0.67 | 7.73 | 1.24 | 1.9 | 1.71 | 1 | -1.01 |
| 0.97 | -2.63 | 1.19 | 1.46 | 9.55 | 5.3 | 1.41 | 0.67 | 7.73 | 1.24 | 1.9 | 2.09 | 1 | -1.18 |
| 0.97 | -2.64 | 1.21 | 0.96 | 8.28 | 5.3 | 1.41 | 0.67 | 7.73 | 1.24 | 1.9 | 1.9 | 1 | -1.12 |
| 0.97 | -2.52 | 1.19 | 0.68 | 7.98 | 5.3 | 1.41 | 0.67 | 7.73 | 1.24 | 1.9 | 2.03 | 1 | -0.9 |
| 0.97 | -2.31 | 1.16 | 0.47 | 8.28 | 5.3 | 1.41 | 0.67 | 7.73 | 1.24 | 1.9 | 2.03 | 1 | -0.78 |
| 0.97 | -2.09 | 1.12 | 0.28 | 8.95 | 5.3 | 1.41 | 0.67 | 7.73 | 1.24 | 1.9 | 2.06 | 1 | -0.79 |
| 0.98 | -2.08 | 1.04 | 0.13 | 12.7 | 5.3 | 1.41 | 0.67 | 7.73 | 1.24 | 1.9 | 2.16 | 1 | -0.67 |
| 0.98 | -4.49 | 1.17 | 3.84 | 22.63 | 5.01 | 1.59 | 0.89 | 7.58 | 1.3 | 1.93 | 1.65 | 2.26 | -0.06 |
| 0.98 | -4.41 | 1.16 | 3.51 | 20.02 | 5.01 | 1.59 | 0.89 | 7.58 | 1.3 | 1.93 | 1.75 | 2.26 | -0.15 |
| 0.98 | -3.73 | 1.2 | 2.66 | 14.4 | 5.01 | 1.59 | 0.89 | 7.58 | 1.3 | 1.93 | 1.92 | 2.26 | -0.14 |
| 0.98 | -4.01 | 1.18 | 2.61 | 15.3 | 5.01 | 1.59 | 0.89 | 7.58 | 1.3 | 1.93 | 1.92 | 2.26 | -0.06 |
| 0.98 | -4.36 | 1.14 | 4.02 | 24.83 | 5.01 | 1.59 | 0.89 | 7.58 | 1.3 | 1.93 | 1.57 | 2.26 | -0.11 |
| 0.97 | -4.42 | 1.21 | 3.68 | 20.94 | 5.01 | 1.59 | 0.89 | 7.58 | 1.3 | 1.93 | 1.64 | 2.26 | -0.13 |
| 0.97 | -4.33 | 1.26 | 3.24 | 16.96 | 5.01 | 1.59 | 0.89 | 7.58 | 1.3 | 1.93 | 1.78 | 2.26 | -0.24 |
| 0.98 | -3.5 | 1.13 | 2.35 | 13.34 | 5.01 | 1.59 | 0.89 | 7.58 | 1.3 | 1.93 | 2.05 | 2.26 | -0.15 |
| 0.98 | -3.92 | 1.1 | 2.38 | 16.87 | 5.01 | 1.59 | 0.89 | 7.58 | 1.3 | 1.93 | 1.93 | 2.26 | -0.09 |
| 0.98 | -4.15 | 1.06 | 4.06 | 27.12 | 5.01 | 1.59 | 0.89 | 7.58 | 1.3 | 1.93 | 1.93 | 2.26 | -0.12 |
| 0.97 | -4.41 | 1.35 | 3.06 | 15.39 | 5.01 | 1.59 | 0.89 | 7.58 | 1.3 | 1.93 | 1.73 | 2.26 | -0.27 |
| 0.98 | -3.55 | 1.23 | 1.96 | 10.78 | 5.01 | 1.59 | 0.89 | 7.58 | 1.3 | 1.93 | 2.08 | 2.26 | -0.23 |
| 0.98 | -3.86 | 1.17 | 1.88 | 13.13 | 5.01 | 1.59 | 0.89 | 7.58 | 1.3 | 1.93 | 2.18 | 2.26 | -0.12 |
| 0.97 | -3.4 | 1.45 | 1.41 | 7.73 | 6.01 | 1.59 | 1.01 | 9.23 | 2.31 | 2.54 | 2.23 | 3.35 | -0.11 |
| 0.96 | -4.01 | 1.47 | 2.63 | 12.8 | 6.01 | 1.59 | 1.01 | 9.23 | 2.31 | 2.54 | 1.83 | 3.35 | -0.1 |
| 0.96 | -3.35 | 1.49 | 1.39 | 6.53 | 6.01 | 1.59 | 1.01 | 9.23 | 2.31 | 2.54 | 2.38 | 3.35 | -0.47 |
| 0.97 | -2.89 | 1.39 | 1.19 | 6.4 | 6.01 | 1.59 | 1.01 | 9.23 | 2.31 | 2.54 | 2.38 | 3.35 | -0.39 |
| 0.97 | -3.19 | 1.32 | 1.25 | 8.65 | 6.01 | 1.59 | 1.01 | 9.23 | 2.31 | 2.54 | 2.25 | 3.35 | -0.18 |
| 0.96 | -3.71 | 1.36 | 2.49 | 13.27 | 6.01 | 1.59 | 1.01 | 9.23 | 2.31 | 2.54 | 1.75 | 3.35 | -0.11 |
| 0.97 | -3.01 | 1.49 | 1.02 | 5.76 | 6.01 | 1.59 | 1.01 | 9.23 | 2.31 | 2.54 | 2.42 | 3.35 | -0.51 |
| 0.97 | -3.18 | 1.41 | 0.92 | 7.01 | 6.01 | 1.59 | 1.01 | 9.23 | 2.31 | 2.54 | 2.54 | 3.35 | -0.26 |
| 0.88 | -1.22 | 1.28 | 1.46 | 7.33 | 5.77 | 1.38 | 0.71 | 7.64 | 1.16 | 1.91 | 1.64 | 1.16 | -1.27 |
| 0.88 | -1.36 | 1.33 | 1.19 | 6.16 | 5.77 | 1.38 | 0.71 | 7.64 | 1.16 | 1.91 | 1.74 | 1.16 | -1.3 |
| 0.87 | -1.43 | 1.36 | 0.9 | 5.19 | 5.77 | 1.38 | 0.71 | 7.64 | 1.16 | 1.91 | 1.78 | 1.16 | -1.36 |
| 0.87 | -1.53 | 1.38 | 0.67 | 4.78 | 5.77 | 1.38 | 0.71 | 7.64 | 1.16 | 1.91 | 1.79 | 1.16 | -1.39 |
| 0.87 | -1.44 | 1.37 | 0.4 | 5.09 | 5.77 | 1.38 | 0.71 | 7.64 | 1.16 | 1.91 | 1.9 | 1.16 | -1.45 |
| 0.87 | -1.32 | 1.33 | 0.36 | 5.23 | 5.77 | 1.38 | 0.71 | 7.64 | 1.16 | 1.91 | 1.91 | 1.16 | -1.52 |
| 0.88 | -1.11 | 1.26 | 0.31 | 6.41 | 5.77 | 1.38 | 0.71 | 7.64 | 1.16 | 1.91 | 1.91 | 1.16 | -1.54 |
| 0.88 | -1.1 | 1.2 | 1.37 | 7.88 | 5.77 | 1.38 | 0.71 | 7.64 | 1.16 | 1.91 | 1.56 | 1.16 | -1.23 |
| 0.88 | -1.19 | 1.27 | 1.23 | 7.16 | 5.77 | 1.38 | 0.71 | 7.64 | 1.16 | 1.91 | 1.63 | 1.16 | -1.33 |
| 0.87 | -1.27 | 1.37 | 1.02 | 6.38 | 5.77 | 1.38 | 0.71 | 7.64 | 1.16 | 1.91 | 1.77 | 1.16 | -1.47 |
| 0.87 | -1.35 | 1.4 | 0.64 | 5.57 | 5.77 | 1.38 | 0.71 | 7.64 | 1.16 | 1.91 | 2.02 | 1.16 | -1.65 |
| 0.87 | -1.35 | 1.36 | 0.03 | 5.36 | 5.77 | 1.38 | 0.71 | 7.64 | 1.16 | 1.91 | 2.03 | 1.16 | -1.87 |
| 0.87 | -1.3 | 1.34 | 0 | 5.4 | 5.77 | 1.38 | 0.71 | 7.64 | 1.16 | 1.91 | 2.07 | 1.16 | -1.73 |
| 0.87 | -1.22 | 1.27 | 0.03 | 5.82 | 5.77 | 1.38 | 0.71 | 7.64 | 1.16 | 1.91 | 2.03 | 1.16 | -1.68 |
| 0.88 | -1.08 | 1.18 | 0.04 | 8.36 | 5.77 | 1.38 | 0.71 | 7.64 | 1.16 | 1.91 | 1.92 | 1.16 | -1.61 |
| 0.88 | -1.35 | 1.43 | 0.96 | 6.34 | 5.77 | 1.38 | 0.71 | 7.64 | 1.16 | 1.91 | 1.72 | 1.16 | -1.44 |
| 0.87 | -1.43 | 1.5 | 0.63 | 5.73 | 5.77 | 1.38 | 0.71 | 7.64 | 1.16 | 1.91 | 2.1 | 1.16 | -1.62 |
| 0.87 | -1.5 | 1.54 | 0.22 | 5.31 | 5.77 | 1.38 | 0.71 | 7.64 | 1.16 | 1.91 | 1.91 | 1.16 | -1.75 |
| 0.87 | -1.5 | 1.53 | -0.13 | 5.45 | 5.77 | 1.38 | 0.71 | 7.64 | 1.16 | 1.91 | 2.03 | 1.16 | -1.87 |
| 0.87 | -1.36 | 1.45 | -0.38 | 6.06 | 5.77 | 1.38 | 0.71 | 7.64 | 1.16 | 1.91 | 2.03 | 1.16 | -1.8 |
| 0.87 | -1.28 | 1.36 | -0.33 | 6.43 | 5.77 | 1.38 | 0.71 | 7.64 | 1.16 | 1.91 | 2.07 | 1.16 | -1.74 |
| 0.87 | -1.08 | 1.24 | -0.31 | 8.68 | 5.77 | 1.38 | 0.71 | 7.64 | 1.16 | 1.91 | 2.17 | 1.16 | -1.71 |
| 0.92 | -2.39 | 1.5 | 1.69 | 7.11 | 5.9 | 1.52 | 0.94 | 8.34 | 0.56 | 2.2 | 1.78 | 2.78 | -0.48 |
| 0.93 | -2.56 | 1.51 | 1.41 | 5.85 | 5.9 | 1.52 | 0.94 | 8.34 | 0.56 | 2.2 | 1.88 | 2.78 | -0.53 |
| 0.91 | -2.53 | 1.54 | 1.17 | 4.95 | 5.9 | 1.52 | 0.94 | 8.34 | 0.56 | 2.2 | 1.93 | 2.78 | -0.61 |
| 0.92 | -2.03 | 1.49 | 0.89 | 4.98 | 5.9 | 1.52 | 0.94 | 8.34 | 0.56 | 2.2 | 1.88 | 2.78 | -0.68 |
| 0.92 | -2.06 | 1.47 | 0.58 | 4.31 | 5.9 | 1.52 | 0.94 | 8.34 | 0.56 | 2.2 | 2.05 | 2.78 | -0.9 |
| 0.92 | -1.9 | 1.43 | 0.55 | 4.25 | 5.9 | 1.52 | 0.94 | 8.34 | 0.56 | 2.2 | 2.07 | 2.78 | -1.05 |
| 0.93 | -1.64 | 1.38 | 0.38 | 4.28 | 5.9 | 1.52 | 0.94 | 8.34 | 0.56 | 2.2 | 2.06 | 2.78 | -1.12 |
| 0.92 | -2.17 | 1.41 | 1.64 | 7.41 | 5.9 | 1.52 | 0.94 | 8.34 | 0.56 | 2.2 | 1.69 | 2.78 | -0.55 |
| 0.92 | -2.32 | 1.55 | 1.6 | 6.93 | 5.9 | 1.52 | 0.94 | 8.34 | 0.56 | 2.2 | 1.76 | 2.78 | -0.62 |
| 0.92 | -2.47 | 1.62 | 1.31 | 5.56 | 5.9 | 1.52 | 0.94 | 8.34 | 0.56 | 2.2 | 1.91 | 2.78 | -0.72 |
| 0.91 | -2.46 | 1.61 | 0.99 | 4.53 | 5.9 | 1.52 | 0.94 | 8.34 | 0.56 | 2.2 | 2.18 | 2.78 | -0.79 |
| 0.91 | -2.16 | 1.51 | 0.49 | 3.74 | 5.9 | 1.52 | 0.94 | 8.34 | 0.56 | 2.2 | 2.2 | 2.78 | -1.09 |
| 0.91 | -1.99 | 1.47 | 0.4 | 3.84 | 5.9 | 1.52 | 0.94 | 8.34 | 0.56 | 2.2 | 2.24 | 2.78 | -1.18 |
| 0.92 | -1.67 | 1.36 | 0.23 | 3.93 | 5.9 | 1.52 | 0.94 | 8.34 | 0.56 | 2.2 | 2.2 | 2.78 | -1.28 |
| 0.93 | -1.4 | 1.28 | -0.02 | 5.13 | 5.9 | 1.52 | 0.94 | 8.34 | 0.56 | 2.2 | 2.08 | 2.78 | -1.16 |
| 0.92 | -1.96 | 1.32 | 1.28 | 8.09 | 5.9 | 1.52 | 0.94 | 8.34 | 0.56 | 2.2 | 1.62 | 2.78 | -0.5 |
| 0.92 | -2.59 | 1.71 | 1.33 | 5.79 | 5.9 | 1.52 | 0.94 | 8.34 | 0.56 | 2.2 | 1.86 | 2.78 | -0.74 |
| 0.91 | -2.67 | 1.75 | 1.02 | 4.7 | 5.9 | 1.52 | 0.94 | 8.34 | 0.56 | 2.2 | 2.27 | 2.78 | -0.8 |
| 0.91 | -2.5 | 1.71 | 0.42 | 3.71 | 5.9 | 1.52 | 0.94 | 8.34 | 0.56 | 2.2 | 2.2 | 2.78 | -1.05 |
| 0.91 | -2.23 | 1.63 | 0.21 | 3.76 | 5.9 | 1.52 | 0.94 | 8.34 | 0.56 | 2.2 | 2.2 | 2.78 | -1.23 |
| 0.92 | -1.82 | 1.49 | 0.03 | 4.01 | 5.9 | 1.52 | 0.94 | 8.34 | 0.56 | 2.2 | 2.24 | 2.78 | -1.33 |
| 0.93 | -1.51 | 1.39 | -0.19 | 5.01 | 5.9 | 1.52 | 0.94 | 8.34 | 0.56 | 2.2 | 2.35 | 2.78 | -1.31 |
| 0.89 | -2.46 | 1.9 | 1.31 | 5.16 | 6.74 | 1.53 | 1.04 | 9 | 2.13 | 2.28 | 1.81 | 3.9 | -0.68 |
| 0.89 | -2.7 | 1.9 | 1.11 | 4.51 | 6.74 | 1.53 | 1.04 | 9 | 2.13 | 2.28 | 1.92 | 3.9 | -0.77 |
| 0.88 | -2.65 | 1.93 | 0.9 | 3.93 | 6.74 | 1.53 | 1.04 | 9 | 2.13 | 2.28 | 1.96 | 3.9 | -0.88 |
| 0.88 | -2.12 | 1.81 | 0.5 | 3.77 | 6.74 | 1.53 | 1.04 | 9 | 2.13 | 2.28 | 2.09 | 3.9 | -1.18 |
| 0.88 | -2.05 | 1.8 | 0.46 | 3.68 | 6.74 | 1.53 | 1.04 | 9 | 2.13 | 2.28 | 2.11 | 3.9 | -1.36 |
| 0.89 | -1.76 | 1.74 | 0.36 | 3.88 | 6.74 | 1.53 | 1.04 | 9 | 2.13 | 2.28 | 2.1 | 3.9 | -1.44 |
| 0.88 | -2.33 | 1.8 | 1.31 | 5.54 | 6.74 | 1.53 | 1.04 | 9 | 2.13 | 2.28 | 1.73 | 3.9 | -0.79 |
| 0.88 | -2.51 | 1.95 | 1.28 | 5.14 | 6.74 | 1.53 | 1.04 | 9 | 2.13 | 2.28 | 1.79 | 3.9 | -0.89 |
| 0.88 | -2.6 | 2.02 | 1.04 | 4.24 | 6.74 | 1.53 | 1.04 | 9 | 2.13 | 2.28 | 1.95 | 3.9 | -1.06 |
| 0.88 | -2.59 | 2.01 | 0.78 | 3.64 | 6.74 | 1.53 | 1.04 | 9 | 2.13 | 2.28 | 2.23 | 3.9 | -1.09 |
| 0.87 | -2.32 | 1.88 | 0.42 | 3.34 | 6.74 | 1.53 | 1.04 | 9 | 2.13 | 2.28 | 2.24 | 3.9 | -1.43 |
| 0.88 | -2.19 | 1.84 | 0.36 | 3.45 | 6.74 | 1.53 | 1.04 | 9 | 2.13 | 2.28 | 2.28 | 3.9 | -1.49 |
| 0.88 | -1.86 | 1.71 | 0.28 | 3.65 | 6.74 | 1.53 | 1.04 | 9 | 2.13 | 2.28 | 2.24 | 3.9 | -1.64 |
| 0.9 | -1.56 | 1.62 | 0.23 | 4.47 | 6.74 | 1.53 | 1.04 | 9 | 2.13 | 2.28 | 2.12 | 3.9 | -1.52 |
| 0.88 | -2.05 | 1.69 | 1.01 | 5.86 | 6.74 | 1.53 | 1.04 | 9 | 2.13 | 2.28 | 1.65 | 3.9 | -0.72 |
| 0.88 | -2.74 | 2.15 | 1.07 | 4.36 | 6.74 | 1.53 | 1.04 | 9 | 2.13 | 2.28 | 1.89 | 3.9 | -1.04 |
| 0.87 | -2.76 | 2.16 | 0.8 | 3.71 | 6.74 | 1.53 | 1.04 | 9 | 2.13 | 2.28 | 2.32 | 3.9 | -1.08 |
| 0.88 | -2.73 | 2.14 | 0.56 | 3.34 | 6.74 | 1.53 | 1.04 | 9 | 2.13 | 2.28 | 2.1 | 3.9 | -1.2 |
| 0.88 | -2.65 | 2.1 | 0.37 | 3.27 | 6.74 | 1.53 | 1.04 | 9 | 2.13 | 2.28 | 2.24 | 3.9 | -1.4 |
| 0.87 | -2.41 | 2.01 | 0.25 | 3.39 | 6.74 | 1.53 | 1.04 | 9 | 2.13 | 2.28 | 2.24 | 3.9 | -1.52 |
| 0.88 | -2.03 | 1.87 | 0.17 | 3.65 | 6.74 | 1.53 | 1.04 | 9 | 2.13 | 2.28 | 2.28 | 3.9 | -1.7 |
| 0.89 | -1.63 | 1.74 | 0.07 | 4.25 | 6.74 | 1.53 | 1.04 | 9 | 2.13 | 2.28 | 2.39 | 3.9 | -1.67 |
| 0.97 | -2.38 | 0.91 | 2.41 | 16.4 | 5.3 | 1.41 | 0.67 | 7.73 | 1.24 | 1.9 | 1.76 | 1 | -0.51 |
| 0.97 | -2.45 | 0.97 | 1.96 | 13.27 | 5.3 | 1.41 | 0.67 | 7.73 | 1.24 | 1.9 | 1.81 | 1 | -0.52 |
| 0.97 | -2.34 | 1 | 1.78 | 12.24 | 5.3 | 1.41 | 0.67 | 7.73 | 1.24 | 1.9 | 1.84 | 1 | -0.65 |
| 0.97 | -2.36 | 1.01 | 1.7 | 12.23 | 5.3 | 1.41 | 0.67 | 7.73 | 1.24 | 1.9 | 1.84 | 1 | -0.66 |
| 0.97 | -2.38 | 1.01 | 1.55 | 12.33 | 5.3 | 1.41 | 0.67 | 7.73 | 1.24 | 1.9 | 1.82 | 1 | -0.63 |
| 0.97 | -2.19 | 1.07 | 1.19 | 9.91 | 5.3 | 1.41 | 0.67 | 7.73 | 1.24 | 1.9 | 1.9 | 1 | -0.63 |
| 0.97 | -2.15 | 1.07 | 1.17 | 9.8 | 5.3 | 1.41 | 0.67 | 7.73 | 1.24 | 1.9 | 1.9 | 1 | -0.66 |
| 0.97 | -2.26 | 1.06 | 1.11 | 10.84 | 5.3 | 1.41 | 0.67 | 7.73 | 1.24 | 1.9 | 1.9 | 1 | -0.63 |
| 0.98 | -2.36 | 1.02 | 0.94 | 13.94 | 5.3 | 1.41 | 0.67 | 7.73 | 1.24 | 1.9 | 1.84 | 1 | -0.7 |
| 0.97 | -2.53 | 1.06 | 0.85 | 15.36 | 5.3 | 1.41 | 0.67 | 7.73 | 1.24 | 1.9 | 1.88 | 1 | -0.72 |
| 0.97 | -2.46 | 1.09 | 0.54 | 14.48 | 5.3 | 1.41 | 0.67 | 7.73 | 1.24 | 1.9 | 1.92 | 1 | -0.8 |
| 0.97 | -2.31 | 0.9 | 2.2 | 15.96 | 5.3 | 1.41 | 0.67 | 7.73 | 1.24 | 1.9 | 1.72 | 1 | -0.49 |
| 0.97 | -2.39 | 0.93 | 2.37 | 15.68 | 5.3 | 1.41 | 0.67 | 7.73 | 1.24 | 1.9 | 1.76 | 1 | -0.56 |
| 0.97 | -2.36 | 0.99 | 1.91 | 12.49 | 5.3 | 1.41 | 0.67 | 7.73 | 1.24 | 1.9 | 1.83 | 1 | -0.51 |
| 0.97 | -2.27 | 1.03 | 1.51 | 10.49 | 5.3 | 1.41 | 0.67 | 7.73 | 1.24 | 1.9 | 1.95 | 1 | -0.48 |
| 0.97 | -2.04 | 1.04 | 1.06 | 9.17 | 5.3 | 1.41 | 0.67 | 7.73 | 1.24 | 1.9 | 1.96 | 1 | -0.67 |
| 0.97 | -1.98 | 1.04 | 0.99 | 9.29 | 5.3 | 1.41 | 0.67 | 7.73 | 1.24 | 1.9 | 1.98 | 1 | -0.72 |
| 0.97 | -1.94 | 1.01 | 0.87 | 9.99 | 5.3 | 1.41 | 0.67 | 7.73 | 1.24 | 1.9 | 1.96 | 1 | -0.7 |
| 0.98 | -2.09 | 0.96 | 0.77 | 13.51 | 5.3 | 1.41 | 0.67 | 7.73 | 1.24 | 1.9 | 1.91 | 1 | -0.7 |
| 0.98 | -2.39 | 0.99 | -0.21 | 29.99 | 5.3 | 1.41 | 0.67 | 7.73 | 1.24 | 1.9 | 1.87 | 1 | -0.75 |
| 0.97 | -2.16 | 0.86 | 2.11 | 16.29 | 5.3 | 1.41 | 0.67 | 7.73 | 1.24 | 1.9 | 1.68 | 1 | -0.6 |
| 0.97 | -2.47 | 1.03 | 1.9 | 12.33 | 5.3 | 1.41 | 0.67 | 7.73 | 1.24 | 1.9 | 1.8 | 1 | -0.49 |
| 0.97 | -2.38 | 1.08 | 1.49 | 10.17 | 5.3 | 1.41 | 0.67 | 7.73 | 1.24 | 1.9 | 1.99 | 1 | -0.46 |
| 0.97 | -2.31 | 1.13 | 1.11 | 8.69 | 5.3 | 1.41 | 0.67 | 7.73 | 1.24 | 1.9 | 1.9 | 1 | -0.49 |
| 0.97 | -2.23 | 1.13 | 0.92 | 8.43 | 5.3 | 1.41 | 0.67 | 7.73 | 1.24 | 1.9 | 1.96 | 1 | -0.65 |
| 0.97 | -2.09 | 1.1 | 0.78 | 8.59 | 5.3 | 1.41 | 0.67 | 7.73 | 1.24 | 1.9 | 1.96 | 1 | -0.72 |
| 0.97 | -1.97 | 1.07 | 0.61 | 9.16 | 5.3 | 1.41 | 0.67 | 7.73 | 1.24 | 1.9 | 1.98 | 1 | -0.74 |
| 0.98 | -2.11 | 1.02 | 0.35 | 11.84 | 5.3 | 1.41 | 0.67 | 7.73 | 1.24 | 1.9 | 2.03 | 1 | -0.72 |
| 0.98 | -2.31 | 0.98 | -0.51 | 27.36 | 5.3 | 1.41 | 0.67 | 7.73 | 1.24 | 1.9 | 1.83 | 1 | -0.73 |
| 0.98 | -2.27 | 0.92 | 0.77 | 19.57 | 5.3 | 1.41 | 0.67 | 7.73 | 1.24 | 1.9 | 1.99 | 1 | -0.8 |
| 0.98 | -4.02 | 1.06 | 3.28 | 20.88 | 5.01 | 1.59 | 0.89 | 7.58 | 1.3 | 1.93 | 1.79 | 2.26 | -0.05 |
| 0.98 | -4.01 | 1.09 | 2.96 | 18.67 | 5.01 | 1.59 | 0.89 | 7.58 | 1.3 | 1.93 | 1.84 | 2.26 | -0.1 |
| 0.98 | -3.98 | 1.13 | 2.88 | 17.91 | 5.01 | 1.59 | 0.89 | 7.58 | 1.3 | 1.93 | 1.86 | 2.26 | -0.12 |
| 0.98 | -4.03 | 1.13 | 2.88 | 18.26 | 5.01 | 1.59 | 0.89 | 7.58 | 1.3 | 1.93 | 1.87 | 2.26 | -0.12 |
| 0.98 | -4.08 | 1.15 | 2.71 | 17.36 | 5.01 | 1.59 | 0.89 | 7.58 | 1.3 | 1.93 | 1.84 | 2.26 | -0.12 |
| 0.98 | -3.9 | 1.19 | 2.32 | 14.44 | 5.01 | 1.59 | 0.89 | 7.58 | 1.3 | 1.93 | 1.92 | 2.26 | -0.11 |
| 0.98 | -3.85 | 1.2 | 2.29 | 14.31 | 5.01 | 1.59 | 0.89 | 7.58 | 1.3 | 1.93 | 1.93 | 2.26 | -0.09 |
| 0.98 | -3.99 | 1.16 | 2.37 | 15.46 | 5.01 | 1.59 | 0.89 | 7.58 | 1.3 | 1.93 | 1.92 | 2.26 | -0.08 |
| 0.98 | -4.1 | 1.14 | 2.28 | 16.76 | 5.01 | 1.59 | 0.89 | 7.58 | 1.3 | 1.93 | 1.86 | 2.26 | -0.11 |
| 0.98 | -4.12 | 1.16 | 2.34 | 17.28 | 5.01 | 1.59 | 0.89 | 7.58 | 1.3 | 1.93 | 1.9 | 2.26 | -0.14 |
| 0.98 | -3.92 | 1.13 | 2.25 | 17.64 | 5.01 | 1.59 | 0.89 | 7.58 | 1.3 | 1.93 | 1.95 | 2.26 | -0.2 |
| 0.98 | -3.89 | 1.14 | 2.86 | 16.72 | 5.01 | 1.59 | 0.89 | 7.58 | 1.3 | 1.93 | 1.85 | 2.26 | -0.07 |
| 0.98 | -3.65 | 1.18 | 2.24 | 13.22 | 5.01 | 1.59 | 0.89 | 7.58 | 1.3 | 1.93 | 1.99 | 2.26 | -0.11 |
| 0.98 | -3.69 | 1.13 | 2.3 | 14.48 | 5.01 | 1.59 | 0.89 | 7.58 | 1.3 | 1.93 | 1.99 | 2.26 | -0.08 |
| 0.98 | -3.92 | 1.1 | 2.38 | 16.87 | 5.01 | 1.59 | 0.89 | 7.58 | 1.3 | 1.93 | 1.93 | 2.26 | -0.09 |
| 0.97 | -3.98 | 1.21 | 2.73 | 15.39 | 5.01 | 1.59 | 0.89 | 7.58 | 1.3 | 1.93 | 1.82 | 2.26 | -0.02 |
| 0.97 | -3.89 | 1.24 | 2.47 | 13.65 | 5.01 | 1.59 | 0.89 | 7.58 | 1.3 | 1.93 | 2.02 | 2.26 | -0.03 |
| 0.98 | -3.74 | 1.24 | 2.1 | 11.95 | 5.01 | 1.59 | 0.89 | 7.58 | 1.3 | 1.93 | 1.99 | 2.26 | -0.1 |
| 0.98 | -3.66 | 1.22 | 2.09 | 12.2 | 5.01 | 1.59 | 0.89 | 7.58 | 1.3 | 1.93 | 1.99 | 2.26 | -0.14 |
| 0.98 | -3.68 | 1.18 | 2.11 | 12.89 | 5.01 | 1.59 | 0.89 | 7.58 | 1.3 | 1.93 | 2 | 2.26 | -0.12 |
| 0.98 | -3.86 | 1.13 | 2.14 | 14.93 | 5.01 | 1.59 | 0.89 | 7.58 | 1.3 | 1.93 | 2.05 | 2.26 | -0.11 |
| 0.97 | -3.34 | 1.48 | 1.09 | 6.97 | 6.01 | 1.59 | 1.01 | 9.23 | 2.31 | 2.54 | 2.38 | 3.35 | -0.17 |
| 0.97 | -3.5 | 1.45 | 1 | 7.66 | 6.01 | 1.59 | 1.01 | 9.23 | 2.31 | 2.54 | 2.31 | 3.35 | -0.24 |
| 0.97 | -3.53 | 1.47 | 1.12 | 8.26 | 6.01 | 1.59 | 1.01 | 9.23 | 2.31 | 2.54 | 2.36 | 3.35 | -0.21 |
| 0.97 | -2.99 | 1.43 | 1.02 | 6.49 | 6.01 | 1.59 | 1.01 | 9.23 | 2.31 | 2.54 | 2.46 | 3.35 | -0.3 |
| 0.97 | -3.17 | 1.37 | 1.07 | 7.67 | 6.01 | 1.59 | 1.01 | 9.23 | 2.31 | 2.54 | 2.39 | 3.35 | -0.21 |
| 0.97 | -3.02 | 1.47 | 0.94 | 6.07 | 6.01 | 1.59 | 1.01 | 9.23 | 2.31 | 2.54 | 2.48 | 3.35 | -0.36 |
| 0.97 | -3.18 | 1.41 | 0.92 | 7.01 | 6.01 | 1.59 | 1.01 | 9.23 | 2.31 | 2.54 | 2.54 | 3.35 | -0.26 |
| 0.88 | -0.94 | 1.18 | 1.08 | 6.7 | 5.77 | 1.38 | 0.71 | 7.64 | 1.16 | 1.91 | 1.77 | 1.16 | -1.47 |
| 0.88 | -1.2 | 1.3 | 0.73 | 5.38 | 5.77 | 1.38 | 0.71 | 7.64 | 1.16 | 1.91 | 1.82 | 1.16 | -1.52 |
| 0.87 | -1.52 | 1.37 | 0.59 | 5.02 | 5.77 | 1.38 | 0.71 | 7.64 | 1.16 | 1.91 | 1.84 | 1.16 | -1.4 |
| 0.87 | -1.31 | 1.33 | 0.46 | 5.11 | 5.77 | 1.38 | 0.71 | 7.64 | 1.16 | 1.91 | 1.85 | 1.16 | -1.53 |
| 0.87 | -1.42 | 1.36 | 0.37 | 5.13 | 5.77 | 1.38 | 0.71 | 7.64 | 1.16 | 1.91 | 1.9 | 1.16 | -1.47 |
| 0.87 | -1.32 | 1.33 | 0.36 | 5.23 | 5.77 | 1.38 | 0.71 | 7.64 | 1.16 | 1.91 | 1.91 | 1.16 | -1.52 |
| 0.87 | -1.19 | 1.29 | 0.34 | 5.79 | 5.77 | 1.38 | 0.71 | 7.64 | 1.16 | 1.91 | 1.91 | 1.16 | -1.52 |
| 0.88 | -1.15 | 1.27 | 0.28 | 7.68 | 5.77 | 1.38 | 0.71 | 7.64 | 1.16 | 1.91 | 1.85 | 1.16 | -1.53 |
| 0.88 | -1.11 | 1.32 | 0.06 | 9.93 | 5.77 | 1.38 | 0.71 | 7.64 | 1.16 | 1.91 | 1.89 | 1.16 | -1.56 |
| 0.88 | -1.19 | 1.38 | -0.2 | 8.89 | 5.77 | 1.38 | 0.71 | 7.64 | 1.16 | 1.91 | 1.93 | 1.16 | -1.54 |
| 0.88 | -0.79 | 1.1 | 1.34 | 8.08 | 5.77 | 1.38 | 0.71 | 7.64 | 1.16 | 1.91 | 1.73 | 1.16 | -1.49 |
| 0.88 | -1.04 | 1.23 | 0.92 | 6.07 | 5.77 | 1.38 | 0.71 | 7.64 | 1.16 | 1.91 | 1.76 | 1.16 | -1.54 |
| 0.87 | -1.49 | 1.34 | 0.74 | 5.63 | 5.77 | 1.38 | 0.71 | 7.64 | 1.16 | 1.91 | 1.84 | 1.16 | -1.35 |
| 0.87 | -1.52 | 1.37 | 0.51 | 5.25 | 5.77 | 1.38 | 0.71 | 7.64 | 1.16 | 1.91 | 1.96 | 1.16 | -1.36 |
| 0.87 | -1.33 | 1.32 | 0.27 | 5.35 | 5.77 | 1.38 | 0.71 | 7.64 | 1.16 | 1.91 | 1.97 | 1.16 | -1.52 |
| 0.87 | -1.3 | 1.32 | 0.26 | 5.41 | 5.77 | 1.38 | 0.71 | 7.64 | 1.16 | 1.91 | 1.99 | 1.16 | -1.56 |
| 0.87 | -1.2 | 1.27 | 0.22 | 6.09 | 5.77 | 1.38 | 0.71 | 7.64 | 1.16 | 1.91 | 1.97 | 1.16 | -1.61 |
| 0.88 | -1.1 | 1.22 | 0.18 | 7.75 | 5.77 | 1.38 | 0.71 | 7.64 | 1.16 | 1.91 | 1.91 | 1.16 | -1.62 |
| 0.89 | -0.99 | 1.22 | -0.48 | 18.37 | 5.77 | 1.38 | 0.71 | 7.64 | 1.16 | 1.91 | 1.88 | 1.16 | -1.63 |
| 0.89 | -0.98 | 1.22 | 0.11 | 9.69 | 5.77 | 1.38 | 0.71 | 7.64 | 1.16 | 1.91 | 1.92 | 1.16 | -1.63 |
| 0.89 | -0.75 | 1.07 | 1.32 | 8.28 | 5.77 | 1.38 | 0.71 | 7.64 | 1.16 | 1.91 | 1.69 | 1.16 | -1.47 |
| 0.87 | -1.54 | 1.4 | 0.76 | 5.63 | 5.77 | 1.38 | 0.71 | 7.64 | 1.16 | 1.91 | 1.81 | 1.16 | -1.33 |
| 0.87 | -1.6 | 1.43 | 0.52 | 5.2 | 5.77 | 1.38 | 0.71 | 7.64 | 1.16 | 1.91 | 2 | 1.16 | -1.34 |
| 0.87 | -1.59 | 1.46 | 0.26 | 4.87 | 5.77 | 1.38 | 0.71 | 7.64 | 1.16 | 1.91 | 1.91 | 1.16 | -1.34 |
| 0.87 | -1.45 | 1.43 | 0.07 | 5.1 | 5.77 | 1.38 | 0.71 | 7.64 | 1.16 | 1.91 | 1.97 | 1.16 | -1.44 |
| 0.87 | -1.3 | 1.37 | 0.03 | 5.6 | 5.77 | 1.38 | 0.71 | 7.64 | 1.16 | 1.91 | 1.97 | 1.16 | -1.58 |
| 0.87 | -1.25 | 1.33 | 0.05 | 6.16 | 5.77 | 1.38 | 0.71 | 7.64 | 1.16 | 1.91 | 1.99 | 1.16 | -1.65 |
| 0.87 | -1.12 | 1.27 | -0.06 | 7.75 | 5.77 | 1.38 | 0.71 | 7.64 | 1.16 | 1.91 | 2.03 | 1.16 | -1.68 |
| 0.89 | -0.97 | 1.21 | -0.69 | 17.49 | 5.77 | 1.38 | 0.71 | 7.64 | 1.16 | 1.91 | 1.84 | 1.16 | -1.68 |
| 0.89 | -0.91 | 1.15 | -0.07 | 12.21 | 5.77 | 1.38 | 0.71 | 7.64 | 1.16 | 1.91 | 2 | 1.16 | -1.65 |
| 0.89 | -1.04 | 1.23 | -0.57 | 15.32 | 5.77 | 1.38 | 0.71 | 7.64 | 1.16 | 1.91 | 1.93 | 1.16 | -1.63 |
| 0.93 | -1.72 | 1.39 | 1.04 | 5.49 | 5.9 | 1.52 | 0.94 | 8.34 | 0.56 | 2.2 | 1.98 | 2.78 | -0.82 |
| 0.93 | -2.27 | 1.51 | 0.78 | 4.33 | 5.9 | 1.52 | 0.94 | 8.34 | 0.56 | 2.2 | 2.03 | 2.78 | -0.94 |
| 0.92 | -2.19 | 1.51 | 0.64 | 4.06 | 5.9 | 1.52 | 0.94 | 8.34 | 0.56 | 2.2 | 2.06 | 2.78 | -0.95 |
| 0.92 | -1.87 | 1.43 | 0.58 | 4.23 | 5.9 | 1.52 | 0.94 | 8.34 | 0.56 | 2.2 | 2.07 | 2.78 | -0.99 |
| 0.93 | -1.75 | 1.41 | 0.53 | 4.39 | 5.9 | 1.52 | 0.94 | 8.34 | 0.56 | 2.2 | 2.04 | 2.78 | -1 |
| 0.92 | -1.95 | 1.48 | 0.35 | 3.8 | 5.9 | 1.52 | 0.94 | 8.34 | 0.56 | 2.2 | 2.12 | 2.78 | -1.07 |
| 0.92 | -1.82 | 1.43 | 0.35 | 3.86 | 5.9 | 1.52 | 0.94 | 8.34 | 0.56 | 2.2 | 2.13 | 2.78 | -1.17 |
| 0.92 | -1.72 | 1.4 | 0.31 | 3.95 | 5.9 | 1.52 | 0.94 | 8.34 | 0.56 | 2.2 | 2.13 | 2.78 | -1.15 |
| 0.93 | -1.77 | 1.38 | 0.16 | 4.96 | 5.9 | 1.52 | 0.94 | 8.34 | 0.56 | 2.2 | 2.06 | 2.78 | -1.04 |
| 0.93 | -1.85 | 1.48 | 0.06 | 6.81 | 5.9 | 1.52 | 0.94 | 8.34 | 0.56 | 2.2 | 2.11 | 2.78 | -1.15 |
| 0.93 | -1.99 | 1.58 | -0.16 | 6.31 | 5.9 | 1.52 | 0.94 | 8.34 | 0.56 | 2.2 | 2.16 | 2.78 | -1.01 |
| 0.93 | -1.51 | 1.31 | 1.45 | 7.46 | 5.9 | 1.52 | 0.94 | 8.34 | 0.56 | 2.2 | 1.93 | 2.78 | -0.82 |
| 0.93 | -2.17 | 1.51 | 1.09 | 5.32 | 5.9 | 1.52 | 0.94 | 8.34 | 0.56 | 2.2 | 1.97 | 2.78 | -0.89 |
| 0.92 | -2.59 | 1.58 | 0.87 | 4.56 | 5.9 | 1.52 | 0.94 | 8.34 | 0.56 | 2.2 | 2.05 | 2.78 | -0.8 |
| 0.92 | -2.36 | 1.56 | 0.64 | 4.07 | 5.9 | 1.52 | 0.94 | 8.34 | 0.56 | 2.2 | 2.19 | 2.78 | -0.94 |
| 0.92 | -1.82 | 1.41 | 0.32 | 3.84 | 5.9 | 1.52 | 0.94 | 8.34 | 0.56 | 2.2 | 2.22 | 2.78 | -1.24 |
| 0.92 | -1.67 | 1.36 | 0.23 | 3.93 | 5.9 | 1.52 | 0.94 | 8.34 | 0.56 | 2.2 | 2.2 | 2.78 | -1.28 |
| 0.93 | -1.53 | 1.29 | 0.15 | 4.68 | 5.9 | 1.52 | 0.94 | 8.34 | 0.56 | 2.2 | 2.14 | 2.78 | -1.24 |
| 0.93 | -1.66 | 1.39 | -0.47 | 13.27 | 5.9 | 1.52 | 0.94 | 8.34 | 0.56 | 2.2 | 2.1 | 2.78 | -1.14 |
| 0.93 | -1.79 | 1.43 | 0.11 | 6.88 | 5.9 | 1.52 | 0.94 | 8.34 | 0.56 | 2.2 | 2.14 | 2.78 | -1.02 |
| 0.93 | -1.48 | 1.25 | 1.59 | 8.21 | 5.9 | 1.52 | 0.94 | 8.34 | 0.56 | 2.2 | 1.89 | 2.78 | -0.8 |
| 0.91 | -2.76 | 1.67 | 0.92 | 4.73 | 5.9 | 1.52 | 0.94 | 8.34 | 0.56 | 2.2 | 2.02 | 2.78 | -0.76 |
| 0.91 | -2.67 | 1.68 | 0.71 | 4.16 | 5.9 | 1.52 | 0.94 | 8.34 | 0.56 | 2.2 | 2.23 | 2.78 | -0.83 |
| 0.92 | -2.45 | 1.65 | 0.47 | 3.74 | 5.9 | 1.52 | 0.94 | 8.34 | 0.56 | 2.2 | 2.13 | 2.78 | -0.93 |
| 0.92 | -2.2 | 1.59 | 0.32 | 3.61 | 5.9 | 1.52 | 0.94 | 8.34 | 0.56 | 2.2 | 2.2 | 2.78 | -1.08 |
| 0.92 | -1.93 | 1.5 | 0.2 | 3.75 | 5.9 | 1.52 | 0.94 | 8.34 | 0.56 | 2.2 | 2.2 | 2.78 | -1.2 |
| 0.92 | -1.72 | 1.42 | 0.13 | 3.96 | 5.9 | 1.52 | 0.94 | 8.34 | 0.56 | 2.2 | 2.22 | 2.78 | -1.31 |
| 0.93 | -1.54 | 1.35 | 0.02 | 4.63 | 5.9 | 1.52 | 0.94 | 8.34 | 0.56 | 2.2 | 2.27 | 2.78 | -1.34 |
| 0.93 | -1.57 | 1.37 | -0.62 | 12.3 | 5.9 | 1.52 | 0.94 | 8.34 | 0.56 | 2.2 | 2.06 | 2.78 | -1.23 |
| 0.93 | -1.59 | 1.33 | -0.11 | 9.09 | 5.9 | 1.52 | 0.94 | 8.34 | 0.56 | 2.2 | 2.23 | 2.78 | -1.04 |
| 0.94 | -1.86 | 1.42 | -0.57 | 11.88 | 5.9 | 1.52 | 0.94 | 8.34 | 0.56 | 2.2 | 2.16 | 2.78 | -0.99 |
| 0.89 | -2 | 1.83 | 0.69 | 4.14 | 6.74 | 1.53 | 1.04 | 9 | 2.13 | 2.28 | 2.03 | 3.9 | -1.23 |
| 0.89 | -2.45 | 1.96 | 0.56 | 3.62 | 6.74 | 1.53 | 1.04 | 9 | 2.13 | 2.28 | 2.09 | 3.9 | -1.31 |
| 0.88 | -2.47 | 1.98 | 0.47 | 3.49 | 6.74 | 1.53 | 1.04 | 9 | 2.13 | 2.28 | 2.12 | 3.9 | -1.37 |
| 0.88 | -2.23 | 1.92 | 0.41 | 3.58 | 6.74 | 1.53 | 1.04 | 9 | 2.13 | 2.28 | 2.12 | 3.9 | -1.39 |
| 0.88 | -2.25 | 1.95 | 0.28 | 3.46 | 6.74 | 1.53 | 1.04 | 9 | 2.13 | 2.28 | 2.18 | 3.9 | -1.44 |
| 0.88 | -2.16 | 1.91 | 0.28 | 3.5 | 6.74 | 1.53 | 1.04 | 9 | 2.13 | 2.28 | 2.19 | 3.9 | -1.51 |
| 0.88 | -2.03 | 1.88 | 0.26 | 3.61 | 6.74 | 1.53 | 1.04 | 9 | 2.13 | 2.28 | 2.19 | 3.9 | -1.44 |
| 0.89 | -2.03 | 1.86 | 0.21 | 4.1 | 6.74 | 1.53 | 1.04 | 9 | 2.13 | 2.28 | 2.12 | 3.9 | -1.38 |
| 0.89 | -2.12 | 1.95 | 0.15 | 4.66 | 6.74 | 1.53 | 1.04 | 9 | 2.13 | 2.28 | 2.17 | 3.9 | -1.48 |
| 0.89 | -2.29 | 2.01 | 0.03 | 4.67 | 6.74 | 1.53 | 1.04 | 9 | 2.13 | 2.28 | 2.22 | 3.9 | -1.52 |
| 0.89 | -1.71 | 1.68 | 0.91 | 5.1 | 6.74 | 1.53 | 1.04 | 9 | 2.13 | 2.28 | 1.98 | 3.9 | -1.24 |
| 0.89 | -2.19 | 1.89 | 0.72 | 3.97 | 6.74 | 1.53 | 1.04 | 9 | 2.13 | 2.28 | 2.02 | 3.9 | -1.33 |
| 0.88 | -2.59 | 2.01 | 0.61 | 3.61 | 6.74 | 1.53 | 1.04 | 9 | 2.13 | 2.28 | 2.11 | 3.9 | -1.2 |
| 0.88 | -2.55 | 2 | 0.48 | 3.41 | 6.74 | 1.53 | 1.04 | 9 | 2.13 | 2.28 | 2.25 | 3.9 | -1.32 |
| 0.88 | -2.22 | 1.91 | 0.28 | 3.39 | 6.74 | 1.53 | 1.04 | 9 | 2.13 | 2.28 | 2.26 | 3.9 | -1.57 |
| 0.88 | -2.12 | 1.87 | 0.25 | 3.5 | 6.74 | 1.53 | 1.04 | 9 | 2.13 | 2.28 | 2.28 | 3.9 | -1.61 |
| 0.88 | -1.97 | 1.81 | 0.22 | 3.6 | 6.74 | 1.53 | 1.04 | 9 | 2.13 | 2.28 | 2.26 | 3.9 | -1.65 |
| 0.89 | -1.79 | 1.74 | 0.18 | 4.01 | 6.74 | 1.53 | 1.04 | 9 | 2.13 | 2.28 | 2.2 | 3.9 | -1.58 |
| 0.89 | -1.89 | 1.81 | -0.17 | 7.59 | 6.74 | 1.53 | 1.04 | 9 | 2.13 | 2.28 | 2.16 | 3.9 | -1.45 |
| 0.9 | -2 | 1.82 | 0.14 | 4.85 | 6.74 | 1.53 | 1.04 | 9 | 2.13 | 2.28 | 2.2 | 3.9 | -1.43 |
| 0.9 | -1.56 | 1.54 | 0.96 | 5.53 | 6.74 | 1.53 | 1.04 | 9 | 2.13 | 2.28 | 1.94 | 3.9 | -1.25 |
| 0.88 | -2.73 | 2.11 | 0.66 | 3.67 | 6.74 | 1.53 | 1.04 | 9 | 2.13 | 2.28 | 2.08 | 3.9 | -1.15 |
| 0.87 | -2.72 | 2.13 | 0.51 | 3.38 | 6.74 | 1.53 | 1.04 | 9 | 2.13 | 2.28 | 2.3 | 3.9 | -1.18 |
| 0.87 | -2.58 | 2.1 | 0.35 | 3.26 | 6.74 | 1.53 | 1.04 | 9 | 2.13 | 2.28 | 2.19 | 3.9 | -1.38 |
| 0.88 | -2.4 | 2.03 | 0.27 | 3.33 | 6.74 | 1.53 | 1.04 | 9 | 2.13 | 2.28 | 2.26 | 3.9 | -1.51 |
| 0.88 | -2.18 | 1.95 | 0.2 | 3.47 | 6.74 | 1.53 | 1.04 | 9 | 2.13 | 2.28 | 2.26 | 3.9 | -1.62 |
| 0.88 | -2.03 | 1.87 | 0.17 | 3.65 | 6.74 | 1.53 | 1.04 | 9 | 2.13 | 2.28 | 2.28 | 3.9 | -1.7 |
| 0.89 | -1.8 | 1.77 | 0.13 | 4.01 | 6.74 | 1.53 | 1.04 | 9 | 2.13 | 2.28 | 2.34 | 3.9 | -1.72 |
| 0.89 | -1.77 | 1.76 | -0.3 | 7.25 | 6.74 | 1.53 | 1.04 | 9 | 2.13 | 2.28 | 2.11 | 3.9 | -1.56 |
| 0.9 | -1.81 | 1.69 | 0.02 | 5.88 | 6.74 | 1.53 | 1.04 | 9 | 2.13 | 2.28 | 2.29 | 3.9 | -1.43 |
| 0.9 | -1.91 | 1.73 | -0.22 | 7.63 | 6.74 | 1.53 | 1.04 | 9 | 2.13 | 2.28 | 2.22 | 3.9 | -1.46 |
